# Geometry-Driven Field-Induced Single-Ion Magnetism in Hexagonal Bipyramidal Tb^3+^ and Ho^3+^ Complexes

**DOI:** 10.1021/acs.inorgchem.5c03348

**Published:** 2025-10-11

**Authors:** Cristina González-Barreira, Paula Oreiro-Martínez, Matilde Fondo, Julio Corredoira-Vázquez, Ana M. García-Deibe, Jesús Sanmartín-Matalobos, Daniel Aravena, Enrique Colacio

**Affiliations:** † Departamento de Química Inorgánica, Facultade de Química, Universidade de Santiago de Compostela, 15782 Santiago de Compostela, Spain; ‡ Institute of Materials (iMATUS), 16780 Universidade de Santiago de Compostela, 15782 Santiago de Compostela, Spain; § Departamento de Química de los Materiales, Facultad de Química y Biología, Universidad de Santiago de Chile, Casilla 40, Correo 33, 9160000 Santiago, Chile; ∥ Departamento de Química Inorgánica, Facultad de Ciencias, 16741 Universidad de Granada, Avda Fuentenueva s/n, 18071 Granada, Spain

## Abstract

The synthesis of the precursors [Ln­(L^N6en^)­(CH_3_COO)_2_]­(BPh_4_)·*n*H_2_O (Ln = Tb, *n* = 0, **1**; Ln = Ho, *n* = 1, **2**·H_2_O), followed by a ligand exchange reaction with triphenylsilanolate, results in the isolation of the complexes {[Ln­(L^N6en^)­(OSiPh_3_)_2_]­(BPh_4_)}·2CH_2_Cl_2_ (Ln = Tb, **3**·2CH_2_Cl_2_; Ln = Ho, **4**·2CH_2_Cl_2_). Single-crystal X-ray diffraction studies of **3**·2CH_2_Cl_2_ and **4**·2CH_2_Cl_2_ revealed that both compounds adopt a hexagonal bipyramidal geometry. Magnetic characterization shows that the complexes behave as single-ion magnets (SIMs) under an optimal applied field of 2000 Oe. Notable, these are the first reported Tb^3+^ and Ho^3+^ complexes with a hexagonal bipyramidal coordination geometry to exhibit such magnet-like behavior. Furthermore, they constitute the first field-induced Tb^3+^ and Ho^3+^ SIMs incorporating a macrocyclic ligand in a nonsandwich topology. Magnetic measurements indicate that the applied field only partially suppresses quantum tunneling of magnetization (QTM) and that at higher temperatures magnetic relaxation is dominated by the Raman process rather than the Orbach mechanism. These experimental observations are supported by ab initio calculations, which provide detailed insights into the electronic structure, including the splitting of f-orbital energy levels, thereby elucidating the origin of the observed magnetic behavior in both cases.

## Graphical abstract

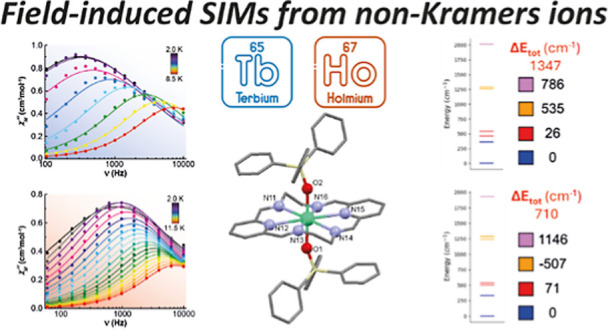

## Introduction

The advancing understanding of factors that enhance the development of air-stable molecular magnets with elevated blocking temperatures,^1,2^ of significant interest in the forefront areas of spintronics^3,4^ and magnetic data storage,^5,6^ has directed the field of molecular magnetism toward the targeted synthesis of mononuclear dysprosium complexes featuring highly axial coordination geometries.^1,2^ Among these, dysprosium-based single-molecule magnets (SMMs) exhibiting hexagonal bipyramidal (hbp) coordination geometries have demonstrated a superior performance, achieving blocking temperatures based on the hysteresis (*T*_B_^H^) up to 40 K in air-stable compounds,^7^ and effective energy barriers (*U*_eff_) of 2427 K.^8^ Despite these significant progresses in the development of molecule magnets based on the Kramers ion Dy^3+^, comparable studies involving non-Kramers ions with high-spin ground states, such as Tb^**3**+^ (*S* = 3) and Ho^3+^ (*S* = 2), and coordination indexes higher than 2 or ligands other than phthalocyanines, remain scarce, especially for holmium­(III). This is the case despite Ho^3+^ ions typically exhibiting the ground multiplet ^5^ *I*_8_, which is particularly appealing due to its high ground-state magnetic moment *m*_J_ = |±8⟩. When this ground state is stabilized alongside a strong axial ligand field, the effective energy barrier (*U*_eff_) can increase substantially. Moreover, the presence of an appropriate symmetry can efficiently suppress quantum tunneling of magnetization (QTM) effects, making Ho^3+^ a promising candidate for designing high-performance single-ion magnets (SIMs).

To date, high-energy barrier Ho^3+^ SIMs have been realized only in compounds with a nearly ideal pentagonal bipyramidal (pbp) geometry, where the equatorial plane consists of weak donor monodentate ligands, such as water or pyridine, with strong donors in axial positions.^9–13^ However, as far as we know, terbium analogues have not shown magnet-like behavior in the absence of an external field.

The synthesis of such highly axial complexes from monodentate ligands is often serendipitous and difficult to predict. Consequently, the use of macrocyclic ligands presents a far more attractive and reliable approach to achieve pbp or hbp geometries. This strategy has been successfully employed in the preparation of high-performance Dy-based SMMs.^7,8,14^ Nevertheless, among Tb^3+^ and Ho^3+^ compounds, only one pbp Ho^3+^ complex synthesized with this approach has been reported, and it was found not to show a SIM-like behavior.^15^ In addition, to the best of our knowledge, no examples of SIMs featuring Tb^3+^ or Ho^3+^ in hexagonal bipyramidal geometries have been described to date.

In this context, and building on our previous success in isolating the hexagonal bipyramidal complex {[Dy­(L^N6en^)­(OSiPh_3_)_2_]­(BPh_4_)}·1.5CH_2_Cl_2_, which has the highest blocking temperature based on the hysteresis observed so far for an air stable molecule magnet,^7^ we present here the structural and magnetic characterization of its holmium and terbium analogues. Our results demonstrate that both compounds function as field-induced single-ion magnets, thereby broadening the scope of coordination geometries capable of supporting slow magnetic relaxation in these non-Kramers lanthanoid ions.

## Experimental Section

### General

All chemical reagents were purchased from commercial sources and used as received without further purification. [Y­(L^N6en^)­(OSiPh_3_)_2_]­(BPh_4_)·3H_2_O was obtained as previously published.^7^

Elemental analyses of C, H, and N were performed on a Thermoscientific Flash Smart analyzer. FT-IR spectra were recorded on a Varian FT-IR 670 spectrometer equipped with an attenuated total reflectance (ATR) attachment in the 500–4000 cm^–1^ range. The lanthanoid and yttrium content of the complexes **3**@Y·1.5CH_2_Cl_2_ and **4**@Y·1.5CH_2_Cl_2_ was determined by inductively coupled plasma optical emission spectroscopy (ICP–MS) using an Agilent 7700 spectrometer.

### Syntheses

#### [Tb­(L^N6en^)­(CH_3_COO)_2_]­(BPh_4_) (1)

2,6-Pyridinedicarboxaldehyde (0.216 g, 1.600 mmol) was dissolved in dry methanol (35 mL). Then, terbium acetate dihydrate (0.298 g, 0.800 mmol) and ethylenediamine (99%, 0.114 mL, 1.680 mmol) were added. After 10 min of stirring, the reaction was refluxed for 24 h, giving an orange suspension. Dry methanol (20 mL) was added to dissolve the solid, and the resultant solution was filtered under vacuum to remove any possible impurity. After that, sodium tetraphenylborate (99%, 0.277 g, 0.800 mmol) was added, and the resultant yellow suspension was stirred for 2 h 30 min. Removing the solvent under a vacuum produced a yellow solid, which was washed with distilled water (100 mL) and centrifuged. The supernatant was decanted, and the yellow solid was dried in a lab stove. Yield: 0.645 g (88%). Elemental analysis Calcd for C_46_H_44_BN_6_O_4_Tb (914.62): C 60.41, N 9.19, H 4.85%. Found: C, 61.11; N, 9.36; H, 4.96%. IR (ATR, **ν̃**/cm^–1^): 1455, 1538 (C=O_OAc_), 1590 (C=N_py_), 1658 (C=N_imine_).

#### [Ho­(L^N6en^)­(CH_3_COO)_2_]­(BPh_4_)·H_2_O (**2**·H_2_O)

2,6-Pyridinedicarboxaldehyde (0.081 g, 0.600 mmol) was dissolved in dry methanol (20 mL). Then, holmium acetate tetrahydrate (0.124 g, 0.300 mmol) and ethylenediamine (99%, 0.043 mL, 0.630 mmol) were added. After 10 min of stirring, the reaction was refluxed for 24 h, giving a yellow orange solution. This solution was filtered under a vacuum to remove any possible impurity. After that, sodium tetraphenylborate (99%, 0.104 g, 0.300 mmol) was added, and the resultant yellow suspension was stirred for 2 h 30 min. Removing the solvent under a vacuum produced a yellow solid, which was washed with distilled water (50 mL) and centrifuged. The supernatant was decanted, and the yellow solid was dried in the lab stove. Yield: 0.236 g (84%). Elemental analysis Calcd for C_46_H_46_BHoN_6_O_5_ (938.64): C 58.86, N 8.95, H 4.94%. Found: C, 58.45; N, 8.82; H, 4.93%. IR (ATR, **ν̃**/cm^–1^): 1456, 1539 (C=O_OAc_), 1590 (C=N_py_), 1660 (C=N_imine_), 3355 (OH).

#### [Tb­(L^N6en^)­(OSiPh_3_)_2_]­(BPh_4_)·2CH_2_Cl_2_ (**3**·2CH_2_Cl_2_)

Triphenylsilanol (98%, 0.062 g, 0.219 mmol) and sodium hydride (60% in a mineral oil dispersion, 0.009 g, 0.219 mmol) were dissolved in dry tetrahydrofuran (5 mL) under an argon atmosphere and stirred for 20 min. Then, this solution was added over **1** (0.100 g, 0.109 mmol) under an argon atmosphere and stirred for 17 h, giving an ochre suspension. The solid was separated by centrifugation and was washed with distilled water (30 mL). The white solid now obtained was dried in a lab stove and recrystallized in dichloromethane by diffusion with diethyl ether at ∼5 °C. This led to the isolation of [Tb­(L^N6en^)­(OSiPh_3_)_2_]­(BPh_4_)·2CH_2_Cl_2_ single crystals, which were dried in a lab stove. Yield: 0.081 g (49%). Elemental analysis Calcd for C_80_H_72_BCl_4_N_6_O_2_Si_2_Tb (1517.14): C, 63.33; N, 5.54; H, 4.78%. Found: C, 63.42; N, 5.49; H, 4.79%. IR (ATR, **ν̃**/cm^–1^): 963 (Si–O), 1593 (C=N_py_), 1662 (C=N_imine_).

#### [Ho­(L^N6en^)­(OSiPh_3_)_2_]­(BPh_4_)·2CH_2_Cl_2_ (**4**·2CH_2_Cl_2_)

Triphenylsilanol (98%, 0.060 g, 0.213 mmol) and sodium hydride (60% in a mineral oil dispersion, 0.009 g, 0.213 mmol) were dissolved in dry tetrahydrofuran (5 mL) under an argon atmosphere and stirred for 20 min. Then, this solution was added over **2**·H_2_O (0.100 g, 0.107 mmol) under an argon atmosphere and stirred for 16 h 30 min, giving an ochre suspension. The solid was separated by centrifugation and washed with distilled water (30 mL). The white solid now obtained was dried in the lab stove and recrystallized in dichloromethane by diffusion with diethyl ether at ∼5 °C. This led to the isolation of [Ho­(L^N6,en^)­(OSiPh_3_)_2_]­(BPh_4_)·2CH_2_Cl_2_ as colorless single crystals, which were dried in a lab stove. Yield: 0.086 g (53%). Elemental analysis Calcd for C_80_H_72_BCl_4_HoN_6_O_2_Si_2_ (1523.19): C, 63.08; N, 5.52; H, 4.76%. Found: C, 63.18; N, 5.49; H, 4.67%. IR (ATR, **ν̃**/cm^–1^): 972 (Si–O), 1593 (C=N_py_), 1664 (C=N_imine_).

#### [Tb_0.1_Y_0.9_(L^N6en^)­(OSiPh_3_)_2_]­(BPh_4_)·1.5CH_2_Cl_2_ (**3@Y**·1.5CH_2_Cl_2_)

**3**·2CH_2_Cl_2_ (0.009 g, 0.006 mmol) and [Y­(L^N6en^)­(OSiPh_3_)_2_]­(BPh_4_)·3H_2_O (0.076 g, 0.057 mmol) were dissolved in dichloromethane (50 mL). The solution was stirred for 24 h at room temperature. Then, the solvent was removed under a vacuum, and the obtained solid was dried in the lab stove. Elemental analysis Calcd for C_79.5_H_71_B Cl_3_N_6_O_2_Si_2_Tb_0.1_Y_0.9_ (1410.31): C, 67.64; N, 5.96; H, 5.03%. Found: C, 68.14; N, 5.90; H, 5.07%. ICP–MS (molar ratio): Tb/Y: 0.1:0.9.

#### [Ho_0.1_Y_0.9_(L^N6en^)­(OSiPh_3_)_2_]­(BPh_4_)·CH_2_Cl_2_ (**4@Y**·CH_2_Cl_2_)

**4**·2CH_2_Cl_2_ (0.009 g, 0.006 mmol) and [Y­(L^N6en^)­(OSiPh_3_)_2_]­(BPh_4_)·3H_2_O (0.076 g, 0.057 mmol) were dissolved in dichloromethane (50 mL). The solution was stirred for 24 h at room temperature. Then, the solvent was removed under a vacuum, and the precipitated solid was dried in the lab stove. Elemental analysis Calcd for C_79_H_70_BCl_2_Ho_0.1_N_6_O_2_Si_2_Y_0.9_ (1368.49): C, 69.27; N, 6.14; H, 5.12%. Found: C, 69.36; N, 6.13; H, 5.18%. ICP–MS (molar ratio): Ho/Y: 0.1:0.9.

### Crystal Structure Analyses

Diffraction data for single crystals of **3**·2CH_2_Cl_2_ and **4**·2CH_2_Cl_2_ were collected at 100(2) K, using monochromatized Mo–*K*α radiation, λ = 0.71073 Å, in a Bruker D8 Venture Photon III-14 diffractometer. Data were routinely processed and corrected, including a multi–scan absorption corrections using the SADABS routine.^16^ The solution of the structure was attained by standard direct methods employing SHELXT^17^ and subsequently refined with the SHELXL program,^18^ using a full matrix least-squares on *F* ^2^. All non–H atoms were refined with anisotropic thermal parameters. Hydrogen atoms were mostly included in the structure factor calculation in geometrically idealized positions, with thermal parameters depending on the parent atom by using a riding model. More details of the refinement, as well as crystal data, are collected in Table S1.

CCDC 2470807 and 2470808 contain the supplementary crystallographic data for this paper. These data can be obtained free of charge from the Cambridge Crystallographic Data Centre via www.ccdc.cam.ac.uk/data_request/cif.

### Powder X-ray Diffraction Studies

The powder diffractograms for **3**·2CH_2_Cl_2_ and **4**·2CH_2_Cl_2_ were recorded in a Philips diffractometer with a control unity type “PW1710”, a vertical goniometer type “PW1820/00”, and a generator type “Enraf Nonius FR590”, operating at 40 kV and 30 mA, using monochromated Cu Kα (λ = 1.5418 Å) radiation. A scan was performed in the range 2 < 2θ < 30° with *t* = 3 s and Δ2θ = 0.02°. LeBail refinement was obtained with the aid of HighScore Plus Version 3.0d.

### Magnetic Measurements

Magnetic measurements for **3**·2CH_2_Cl_2_ and **4**·2CH_2_Cl_2_ were carried out with a DynaCool-9T Physical Property Measurement System (PPMS) instrument. The direct-current (*dc*) magnetic susceptibility data were recorded under a magnetic field of 0.1 T in the temperature range of 2–300 K. Magnetization measurements at 2.0 K were recorded under magnetic fields ranging from 0 to 7 T. Diamagnetic corrections were estimated from Pascal’s Tables. Alternating current (*ac*) susceptibility measurements at different fields were performed with an oscillating *ac* field of 3 Oe and ac frequency of 10,000 Hz. *ac* measurements under an applied field of 2000 Oe were recorded with *ac* frequencies in the range 60–10,000 Hz.

### Computational Details

CASSCF calculations were performed using the ORCA 5.0.3 software package.^19^ The active space consisted of the seven 4f electrons, resulting in CASSCF­(8,7) for Tb^3+^ and CASSCF­(10,7) for Ho^3+^. The number of roots considered all possible f–f excitations and included 7 heptuplets, 140 quintets, 588 triplets, and 490 singlets for Tb^3+^ and 35 quintets, 210 triplets, and 196 singlets for Ho^3+^. Molecular geometries were directly obtained from the crystallographic information files and were based on the metal centers labeled “Tb1” and “Ho1” from **3**·2CH_2_Cl_2_ and **4**·2CH_2_Cl_2_, respectively. The basis set for lanthanoid ions was SARC2-DKH-QZVP^20^ while light elements were described using Def2-TZVP.^21^ Scalar relativistic effects were described by the DKH2 Hamiltonian^22^ and spin–orbit coupling was incorporated using a quasi-degenerate perturbation theory step. f-orbital energies were calculated using ab initio ligand field theory (AILFT)^23^ and their contributions to the ground multiplet splitting were analyzed using a ligand field stabilization energy (LFSE) approximation.^24^

## Results and Discussion

### Synthesis and Structural Characterization

{[Ln­(L^N6en^)­(OSiPh_3_)_2_]­(BPh_4_)}·2CH_2_Cl_2_ (Ln = Tb; Ln = Ho) were isolated from the precursors [Ln­(L^N6en^)­(CH_3_COO)_2_]­(BPh_4_)·*n*H_2_O (Ln = Tb, *n* = 0, **1**; Ln = Ho, *n* = 1, **2**·H_2_O). These precursors were synthesized following a procedure previously reported (Figure 1).^14,25^ The subsequent replacement of the auxiliary acetate ligands with triphenylsilanolate was carried out using a modified version of a published method,^7,26,27^ as outlined in Figure 1. The recrystallization of objective solids **3** and **4** by the diffusion of diethyl ether into a dichloromethane solution of the complexes at *ca*. 5 °C allowed the collection of colorless single crystals of {[Tb­(L^N6en^)­(OSiPh_3_)_2_]­(BPh_4_)}·2CH_2_Cl_2_ (**3**·2CH_2_Cl_2_) and {[Ho­(L^N6en^)­(OSiPh_3_)_2_]­(BPh_4_)}·2CH_2_Cl_2_ (**4**·2CH_2_Cl_2_), respectively. These complexes are stable in air, as no significant changes were observed in their aspect or experimental elemental analyses after one year.

**Figure 1:**
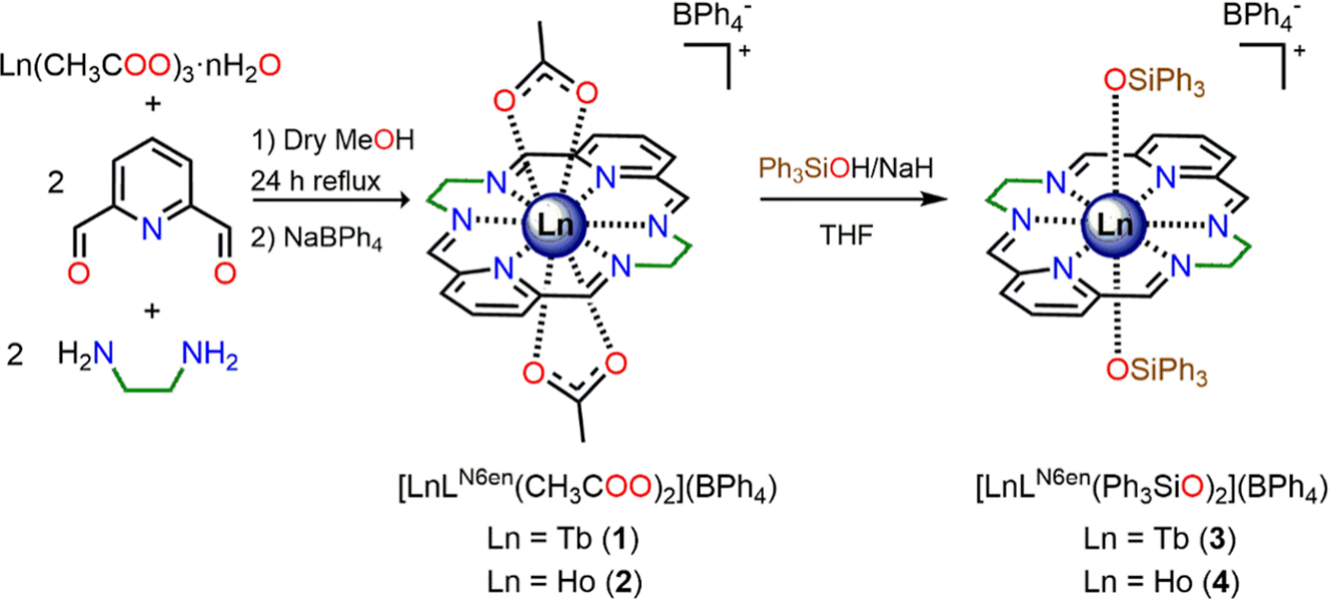
Reaction scheme for the isolation of the precursors **1** and **2** and the objective complexes **3** and **4**. Solvate molecules are omitted for clarity.

The diluted {[Ln_0.1_Y_0.9_(L^N6en^)­(OSiPh_3_)_2_]­(BPh_4_)}·nCH_2_Cl_2_ (Ln = Tb, *n* = 1.5, **3@Y**·1.5CH_2_Cl_2_; Ln = Ho, *n* = 1, **4@Y**·CH_2_Cl_2_) complexes were similarly obtained, by mixing the lanthanoid {[Ln­(L^N6en^)­(OSiPh_3_)_2_]­(BPh_4_)}·2CH_2_Cl_2_ and reported [Y­(L^N6en^)­(OSiPh_3_)_2_]­(BPh_4_)·3H_2_O^7^ species in the adequate ratios.

Complexes **1**, **2**·3H_2_O, **3**·2CH_2_Cl_2_, and **4**·2CH_2_Cl_2_ were characterized by elemental analyses and IR spectroscopy. In addition, **3**·2CH_2_Cl_2_ and **4**·2CH_2_Cl_2_ were also analyzed by single and powder X-ray diffraction techniques.

The comparison of the IR spectra of the precursors **1** and **2**·3H_2_O with those of **3**·2CH_2_Cl_2_ and **4**·2CH_2_Cl_2_ clearly agree with the total displacement of the acetate donors by triphenylsilanolate ones (Figure S1). This is shown by the disappearance in the spectra of the final products of the bands at *ca*. 1540 and 1455 cm^–1^ present in the precursors, which are assigned to CO vibrations of the acetate group^25^ and by the appearance of a new intense band at *ca*. 970 cm^–1^, assigned to the Si–O stretching frequency.^28^

The diluted compounds **3@Y**·1.5CH_2_Cl_2_ and **4@Y**·CH_2_Cl_2_ were characterized by elemental analysis and ICP–MS measurements, which agree with the proposed formulations, with an Ln/Y ratio of 0.1:0.9. In addition, they were also characterized by IR spectroscopy. The comparison of the IR spectra of each diluted sample with the corresponding undiluted one shows overlaid spectra (Figure S2). Accordingly, this indicates that the structures of the undiluted and diluted species are very similar.

### X-ray Diffraction Studies

The main experimental data for the single X-ray diffraction studies of **3**·2CH_2_Cl_2_ and **4**·2CH_2_Cl_2_ are summarized in Table S1.

The compounds are closely related and will be discussed together, although their symmetry is a bit different. Thus, the asymmetric unit of **3**·2CH_2_Cl_2_ contains two crystallographically different, but chemically equivalent, halves of the cationic complex [Tb­(L^N6en^)­(OSiPh_3_)_2_]^+^, with the metal atoms located on inversion centers, as well as a complete [BPh_4_]^−^ anion. Consequently, the two whole complex molecules, which will be called **3a** and **3b**, are related by the symmetry operation –*x*, −*y*, −*z*. For cation **3b** (which contains Tb2), the carbon and nitrogen atoms of the imine and ethylene chain are disordered over two sites (occupation sites 0.535 (**3b1**):0.465 (**3b2**). Additionally, some of the carbon atoms of one triphenylsilanolate ligand are also disordered. In the case of **4**·2CH_2_Cl_2_, the asymmetric unit contains two crystallograhically independent [Ho­(L^N6en^)­(OSiPh_3_)_2_]^+^ cations, which are not generated by symmetry operations, and two [BPh_4_]^−^ anions. These two molecules will be termed **4a** and **4b**. The whole **4b** cation is disordered over two sites (occupation sites 0.525­(**4b1**):0.475­(**4b2**)), except for the triphenylsilanote ligands, which remain ordered.

Accordingly, despite the presence of disorder and differences in symmetry, both complexes contain two crystallographically distinct yet chemically equivalent [Ln­(L^N6en^)­(OSiPh_3_)_2_]­(BPh_4_) molecules in addition to two dichloromethane solvates per formula unit. Representations of the cationic species are shown in Figure 2, S3 and S4, and their main distances and angles are summarized in Table S2.

**Figure 2:**
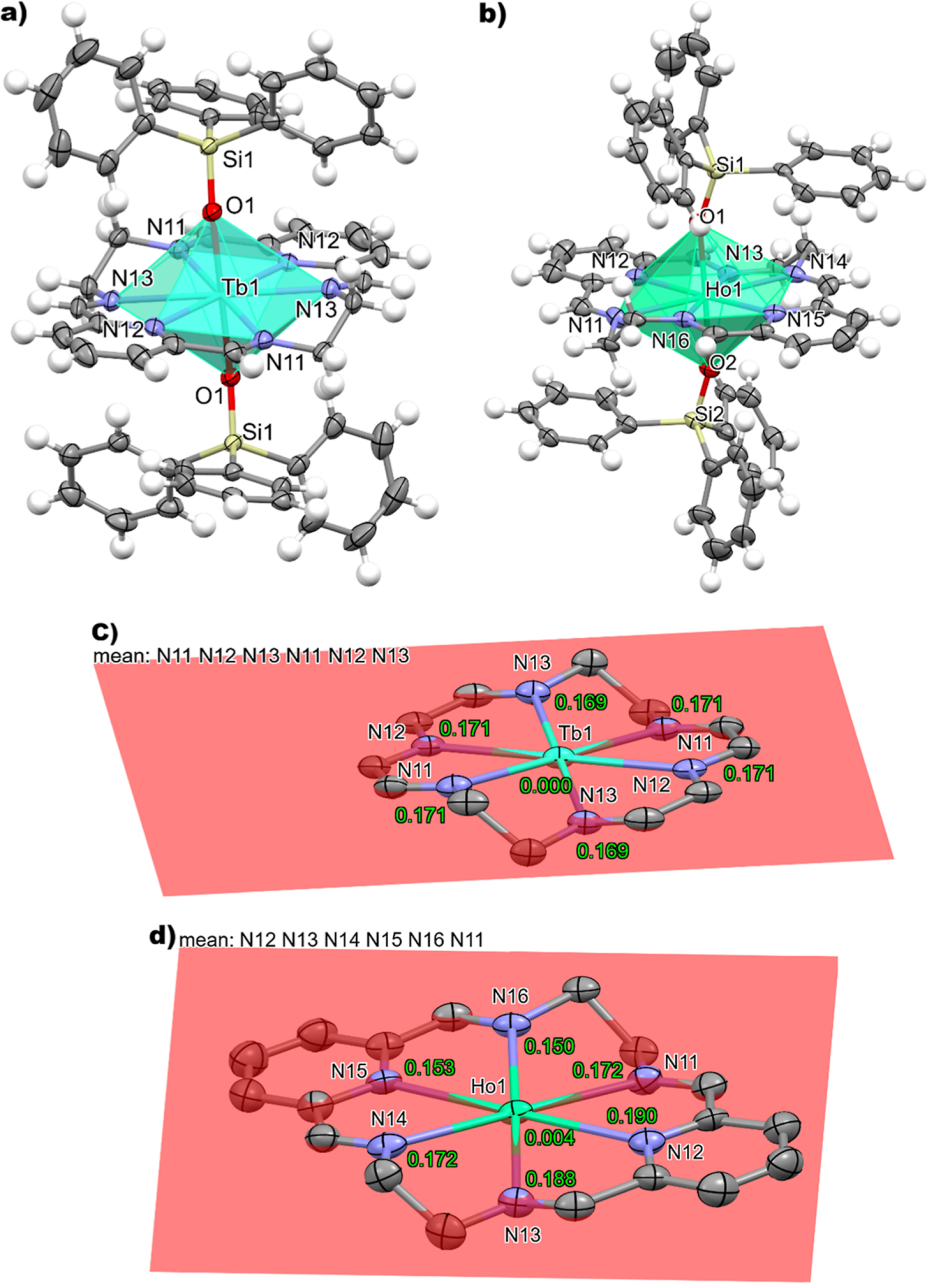
Ellipsoids diagram for the cation [Ln­(L^N6en^)­(OSiPh_3_)_2_]^+^ in the {[Ln­(L^N6en^)­(OSiPh_3_)_2_]­(BPh_4_)} complexes **3a** (a) or **4a** (b), showing Tb1 or Ho1 as a polyhedron. Only heteroatoms of the asymmetric unit and those corresponding to the coordination sphere are labeled. The *N*_6_ equatorial environment of Tb1 in **3a** (c) or Ho1 in **4a** (d), showing the deviation from the mean *N*_6_ calculated plane.

The cationic complexes [Ln­(L^N6en^)­(OSiPh_3_)_2_]^+^ displays an *N*_6_ *O*_2_ coordination environment, composed of the 6 nitrogen atoms from the macrocycle and two apical triphenylsilanote donors. This environment is similar to those observed for other octacoordinated dysprosium complexes derived from *N*_6_ macrocycles.^14^

Calculations of the degree of distortion of the *LnN*_6_ *O*_2_ core relative to an ideal polyhedron of eight vertexes with the SHAPE program^29^ (Table S3), reveal a hexagonal bipyramidal geometry for both compounds. Thus, it is worth noting that these are the first hbp terbium and holmium complexes obtained from such *N*_6_ macrocyclic ligands (Table S4).

The neutral *N*_6_ donor lies in the equatorial plane, with both triphenylsilanolate anions occupying opposite apical positions, with a perfect O–Ln–O angle of 180° for **3**·2CH_2_Cl_2_ and a less open angle for **4**·2CH_2_Cl_2_, of 178.6(2)° for Ho1 and 171.4(6)° for Ho2. The lower distortion of the polyhedron in **3**·2CH_2_Cl_2_ is also shown by the calculated plane formed by the six *N*-donor atoms. In this complex, the maximum deviation of the nitrogen atoms from the least-squares plane is *ca*. 0.17 Å for **3a** (*ca*. 0.079 Å for **3b1** and 0.04 Å for **3b2**), with Tb^3+^ being contained in the plane. In the case of **4**·2CH_2_Cl_2_, the maximum deviation of any atom from the *N*_6_ plane is shown by **4b1** (ca. 0.43 Å, with Ho1 deviating *ca*. 0.014 Å from the plane). In **4b2**, this maximum deviation is *ca*. 0.37, with the Ho2′ atom protruding 0.001 Å from the plane, while for **4a**, the maximum N atom deviation is *ca* 0.19 Å and Ho1 lies just *ca*. 0.004 Å above the plane. The less deviation from the ideal hbp geometry for **3**·2CH_2_Cl_2_ is also confirmed by the ChSM values, which oscillate between 1.124 and 1.687, while for **4**·2CH_2_Cl_2_ range from 1.171 to 2.009 (Table S3).

When the Ln–donor distances and angles of these compounds (Table S2) are compared with those reported for Tb^3+^ and Ho^3+^ complexes based on *N*_6_ macrocycles without pendant arms (Table S4), they are within the usual ranges. However, it is noteworthy that the Ho–N distances in **4**·2CH_2_Cl_2_ are significantly longer (exceeding 2.6 Å) than those observed in the only reported holmium complex featuring a pyridine-based macrocycle and triphenylsilanolate as auxiliary ligands (Ho–N bonds ca. 2.5 Å or shorter)^15^ and than in the other eight-coordinated complexes.^30^ This former related complex with a macrocycle donor adopts a pentagonal bipyramidal geometry, while **4**·2CH_2_Cl_2_ displays a hbp environment for Ho^3+^, which appears to weaken the equatorial ligand field compared to the pentagonal bipyramidal arrangement. Besides, the average Ho–O distance in **4**·2CH_2_Cl_2_ is 2.13 Å, while in the published pbp complex it is 2.16 Å, pointing to a weaker axial field.

The packing diagrams of neighboring molecules in the crystal structures of both complexes reveal that the lanthanoid ions are well isolated, with the shortest Ln^3+^···Ln^3+^ separations being *ca*. 11.34 Å in both cases.

Powder X-ray diffraction measurements for both compounds (Figure S5) were also performed, and these reveal that the isolated products were obtained with high purity as no additional peaks were observed in the experimental diffractogram.

### Magnetic Properties

***dc*** magnetic susceptibility measurements were recorded for **3**·2CH_2_Cl_2_ and **4**·2CH_2_Cl_2_ as functions of the temperature. The graphs of χ_M_ *T* vs *T* (Figure S6) show χ_M_ *T* values at 300 K of 11.6 and 14.5 cm^3^ mol^–1^ K for **3**·2CH_2_Cl_2_ and **4**·2CH_2_Cl_2_, respectively, which are very close to the expected ones for one uncoupled Tb^3+^ ion with a ^7^F_6_ ground state (11.82 cm^3^ mol^–1^ K) or Ho^3+^ ion with a ^5^I_8_ ground state (14.07 cm^3^ mol^–1^ K) at room temperature. The experimental curves continuously decrease until 2 K, reaching χ_M_ *T* values close to 9.3 and 12.2 cm^3^·mol^–1^ K. This drop arises from the split of the ground multiplet by crystal field effects.

Field-dependent magnetization at 2 K at a maximum applied field of 7 T tends to be 4.6 Nμ_B_ for **3**·2CH_2_Cl_2_ and 5.9 Nμ_B_ for **4**·2CH_2_Cl_2_ (Figure S6). These values are lower than the theoretical saturation characteristic of an isolated Tb^3+^ (9 Nμ_B_) or Ho^3+^ (10 Nμ_B_) ion, which agree with highly anisotropic Ln^3+^ ions.

The dynamic magnetic properties of the two mononuclear complexes were also studied, but none of the compounds shows peaks of the out-of-phase susceptibility (χ_M_^″^) as a function of the temperature or the frequency above 2 K, contrary to what happens with the dysprosium analogue {[Dy­(L^N6en^)­(OSiPh_3_)_2_]­(BPh_4_)}·1.5CH_2_Cl_2_.^7^ This lack of SMM behavior can be a consequence of the non-Kramer nature of the Ho^3+^ (suggested *m*_J_ ground state ± 8) and Tb^3+^ (suggested *m*_J_ ground state ± 6) ions, which are prone to efficient quantum tunneling of magnetization (QTM). In these ions, the ground-state doublet can be split by small transverse crystal field components, creating a pair of nearly degenerate states. This can suppress slow magnetic relaxation even in systems with significant magnetic anisotropy, such as **3**·2CH_2_Cl_2_ and **4**·2CH_2_Cl_2_.

Accordingly, alternating-current (*ac*) data were newly recorded in the presence of an optimum field of 2000 Oe (Figure S7) and now χ_M_^″^ for **3**·2CH_2_Cl_2_ and **4**·2CH_2_Cl_2_ shows frequency and temperature dependence in the range 2–8.5 K for **3**·2CH_2_Cl_2_ and 2–11.5 K for **4**·2CH_2_Cl_2_, with clear peaks (Figures 3 and S8). This agrees with the existence of a field-induced relaxation. Besides, the Cole–Cole plots (Figure S9) display curves with α (distribution parameter) values in the range 0.42–0.05 for **3**·2CH_2_Cl_2_ and 0.43–0.07 for **4**·2CH_2_Cl_2_. The distribution parameter α corresponds to a single relaxation process when α = 0, while increasing α values reflect a broader distribution of relaxation times.

**Figure 3:**
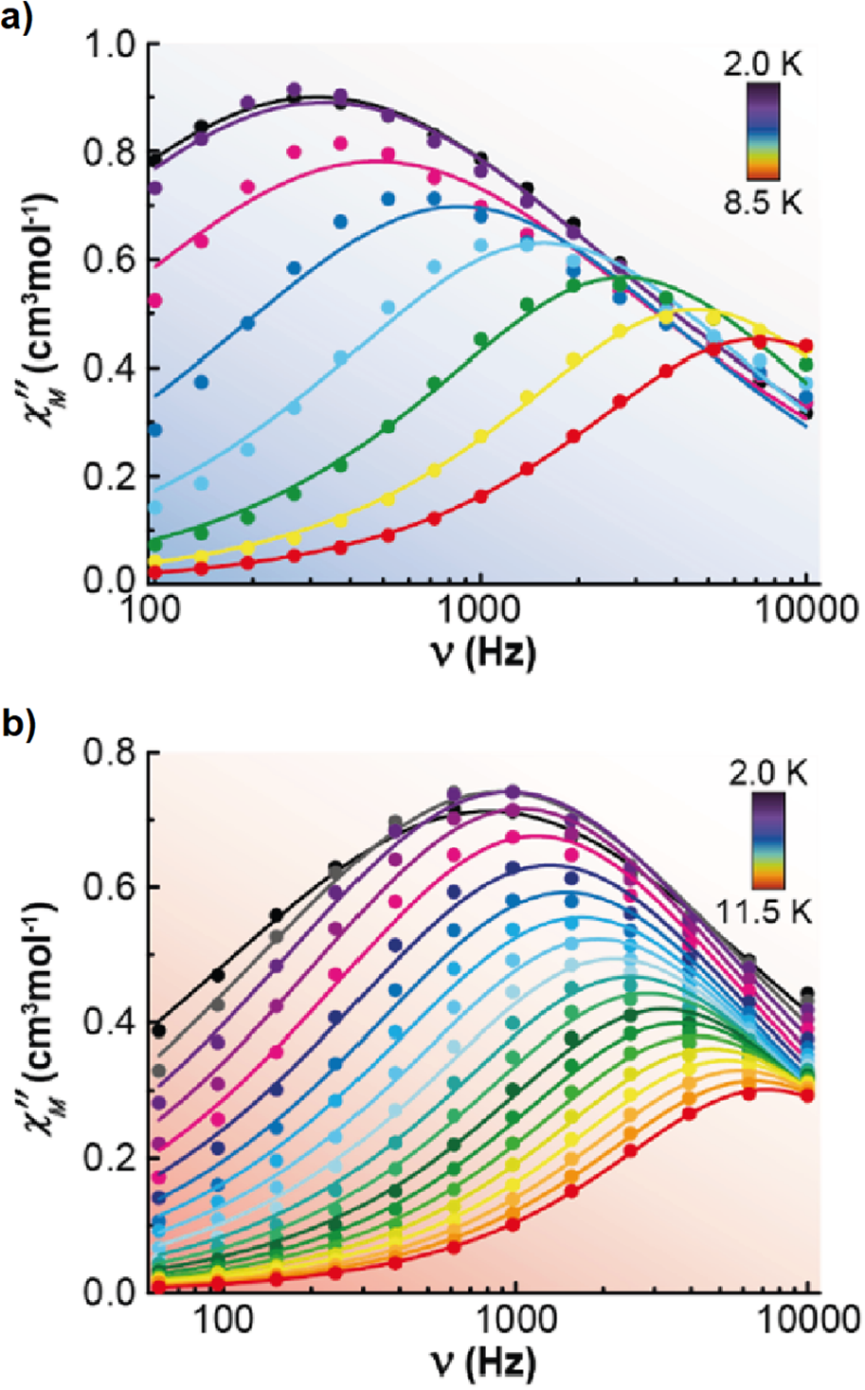
Frequency dependence of χ_M_^″^ for **3**·2CH_2_Cl_2_ (a) and **4**·2CH_2_Cl_2_ (b) in a *dc*-applied field of 2000 Oe at different temperatures.

The relaxation dynamics were examined by the temperature dependence of the relaxation time (Figure 4). We tried to fit these plots considering all of the possible relaxation processes (Orbach, Raman, direct, and QTM), according to eq 1, individually or grouped.

1 $$\tau^{-1}={\tau_{0}}^{-1}e^{-U_{eff}/k_{B}T}+{CT}^{n}+AT+{\tau_{QTM}}^{-1}$$

The best fits of the data (Figure 4) are obtained in both cases considering Raman and QTM relaxation processes, and these render the parameters *C* = 6.2(11) s^–1^ K^–*n*^, *n* = 4.1(1), and τ_QTM_ = 6.1(6) × 10^–4^ s for **3**·2CH_2_Cl_2_ and *C* = 62.0(20) s^–1^ K^–*n*^, *n* = 2.6(1), and τ_QTM_ = 2.4(1) × 10^–4^ s for **4**·2CH_2_Cl_2_.

These data show that the QTM is only partially suppressed by the external field, in agreement with the χ_M_^″^ vs *T* graphs (Figure S8). In addition, the results deviate from expectations, as Tb^3+^ ions are generally associated with higher effective energy barriers compared to their Ho^3+^ analogues and, thus, are anticipated to exhibit a superior magnetic performance. However, the magnetic behavior observed is quite similar for both complexes, with QTM relaxation proceeding slightly faster in the Ho^3+^ compound. Notably, neither system displays clear evidence of Orbach relaxation.

**Figure 4:**
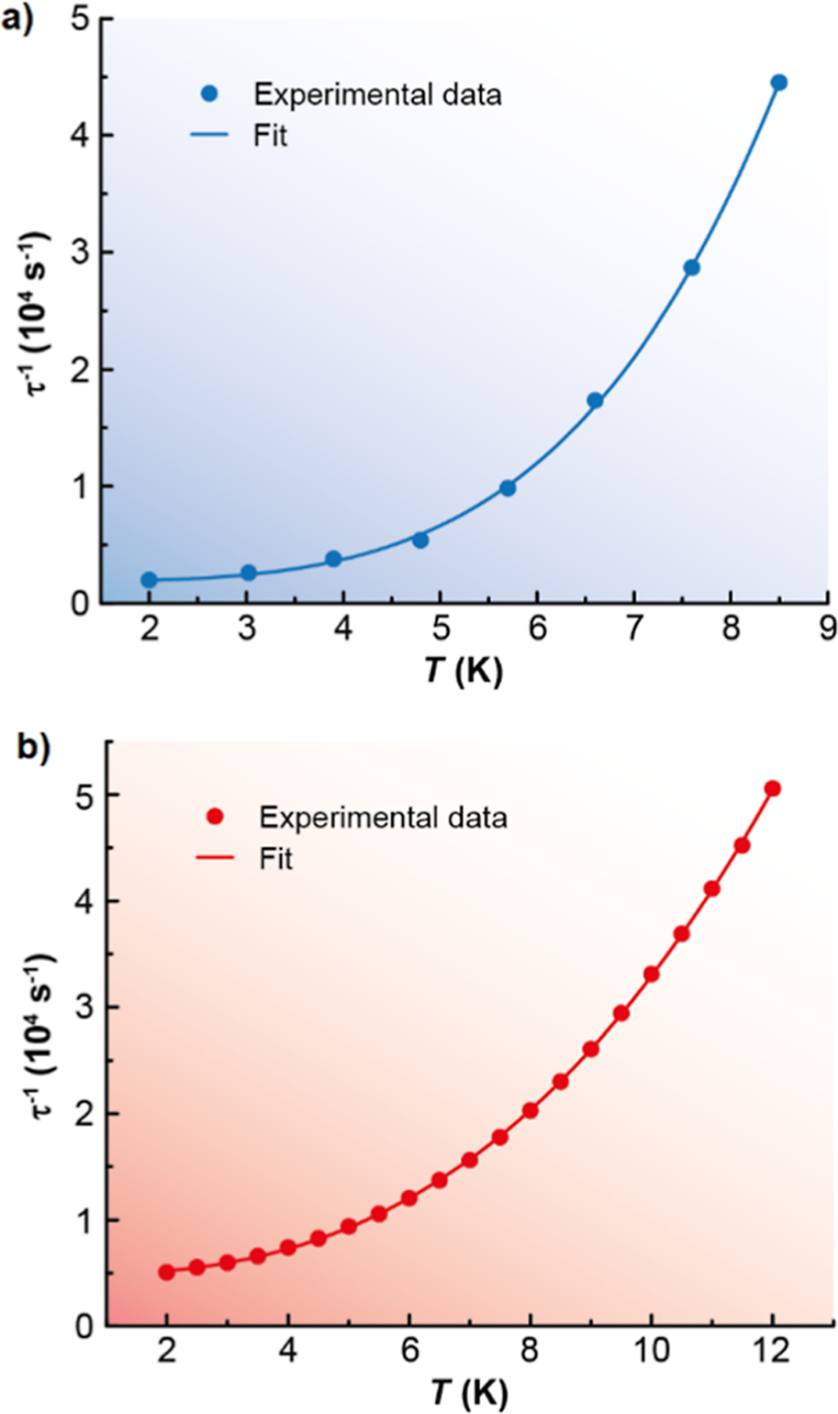
Dependence of the relaxation time with the temperature for **3**·2CH_2_Cl_2_ (a) and **4**·2CH_2_Cl_2_ (b) in a dc applied field of 2000 Oe. The blue and red solid lines accounts for the best fit (*r* ^2^ > 0.99) considering Raman plus QTM relaxation processes.

Attempts to fit the curves considering an Orbach process led to a worse fit, and *U*_eff_ values close to 30 K for both complexes. The energy barrier between the ground and first excited states for these compounds was also computed by ab initio calculations (*ca*. 130 cm^–1^ for **3**·2CH_2_Cl_2_ and 430 cm^–1^ for **4**·2CH_2_Cl_2_, vide infra), and the disparity between the obtained barrier from experimental magnetic data and from calculations also supports the non-Orbach relaxation pathway.

It should also be noted that the *C* value observed for **4**·2CH_2_Cl_2_ may appear relatively high but is not unprecedented. Indeed, values as large as 357 s^–1^ K^–*n*^ have been reported for other field-induced SMMs.^31^ Attempts to fit the data for this holmium complex by including the direct process and/or by constraining the *C* parameter to lower values were unsuccessful and resulted in poorer fits.

In a new effort to fully suppress the quantum tunneling of the magnetization and reveal the intrinsic energy barrier, the complexes were diluted with yttrium. New *ac* susceptibility measurements were performed for {[Tb_0.1_Y_0.9_(L^N6en^)­(OSiPh_3_)_2_]­(BPh_4_)}·1.5CH_2_Cl_2_ (**3@Y**·1.5CH_2_Cl_2_) and {[Ho_0.1_Y_0.9_(L^N6en^)­(OSiPh_3_)_2_]­(BPh_4_)}·CH_2_Cl_2_ (**4@Y**·CH_2_Cl_2_). These measurements, recorded at 10,000 Hz under various applied magnetic fields (Figure S10), confirm that dilution does not lead to the improvement of the magnetic relaxation behavior compared to the undiluted samples. Therefore, these results clearly indicate that intermolecular dipolar interactions are not the primary cause of the observed QTM.

Accordingly, although none of the complexes behave as SIMs in the absence of an external magnetic field, they represent the first examples of Tb^3+^ and Ho^3+^ complexes with a hexagonal bipyramidal geometry to exhibit a SMM-like behavior under an applied field. Notably, they are also the first field-induced SIMs based on Tb^3+^ and Ho^3+^ ions coordinated by an *N*_6_ macrocyclic ligand or by macrocyclic ligands in a nonsandwich arrangement. Therefore, to gain deeper insights into their magnetic properties, ab initio calculations were carried out.

### Ab initio Calculations

The magnetic anisotropy of the Tb^3+^ and Ho^3+^ complexes **3**·2CH_2_Cl_2_ and **4**·2CH_2_Cl_2_ was further analyzed by means of CASSCF­(*n*,7) calculations,^32^ as implemented in the ORCA 5.0.3 program.^19,33^ The f-orbital energies obtained from the ab initio ligand field approach are similar for both molecules, in line with their comparable coordination environments. Thus, only the orbitals for the Ho^3+^ ion are shown in Figure 5. As expected, the most destabilized f-orbital is f_*z*_^3^ (or f_0_), as it points directly to the highly repulsive axial ligands. Its energy differs only slightly between the Tb^3+^ (2011 cm^–1^) and Ho^3+^ (1927 cm^–1^) complexes (Table 1). The remaining orbitals order roughly in pairs, depending on the relative orientation of their lobes with respect to the *z*-axis (Figure 5). The next block (f_±1_) has an energy around 1270 cm^–1^ and a splitting close to 40 cm^–1^ (see Table 1), which is small due to the high symmetry of the coordination environment.

**Table 1:** AILFT Energies (cm^–1^) for the 4f orbitals of Tb^3+^ and Ho^3+^ Complexes

	Tb^3+^		Ho^3+^	
orbital block	*E*_orb_ (cm^–1^)	block average	*E*_orb_ (cm^–1^)	block average
f_±3_	0.0	182.2	0.0	163.5
	364.3		327.0	
f_±2_	460.9	501.3	503.3	518.7
	541.7		534.0	
f_±1_	1256.5	1273.9	1243.7	1265.2
	1291.2		1286.6	
f_0_	2010.5	2010.5	1927.1	1927.1

The f_±2_ orbital pair lies around 500 cm^–1^, with a splitting comparable to that of f_±1_. Finally, the f_±3_ orbitals are oriented in the *xy* plane and display a much larger splitting (364 or 327 cm^–1^), as one of these orbitals ( $f_{x\left(x^{2}-3y^{2}\right)}$ ) points directly to the six N-donor atoms of the macrocycle and the other ( $f_{y\left({3x}^{2}-y^{2}\right)}$ ) points along the bisection of the N-Ln-N angles.

**Figure 5:**
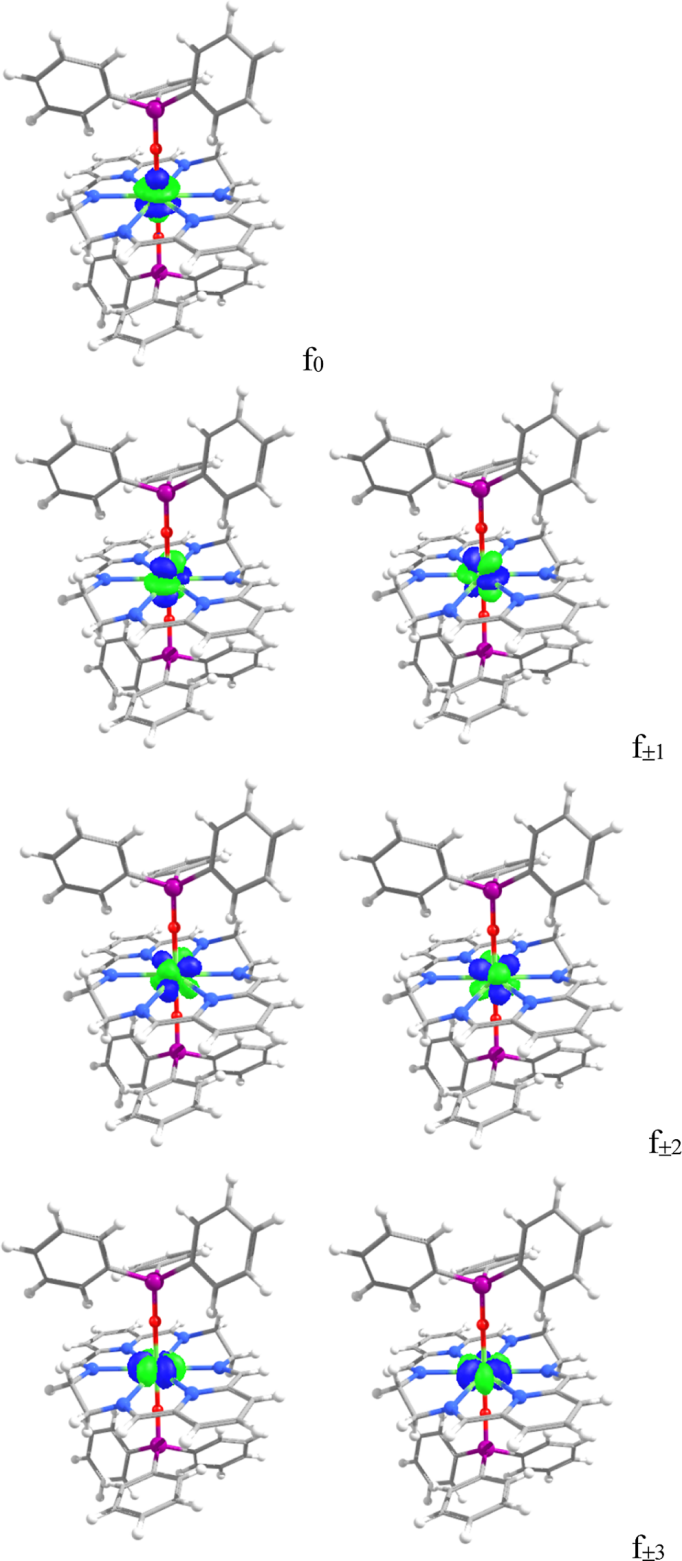
Orbitals for Ho^3+^ in the cation [Ho­(L^N6en^)­(OSiPh_3_)_2_]^+^.

As the f-orbitals split into an axial pattern, it is possible to estimate the energies of the ground multiplet states using a single LFSE summation.

Based on the coefficients for Ho^3+^ and Tb^3+^ presented in ref 23, we estimated the splitting of the ground multiplet (Δ*E*_tot_) decomposing contributions of each orbital block. Figure 6 presents a comparison between CASSCF and LFSE results, which exhibit a close agreement for both complexes.

**Figure 6:**
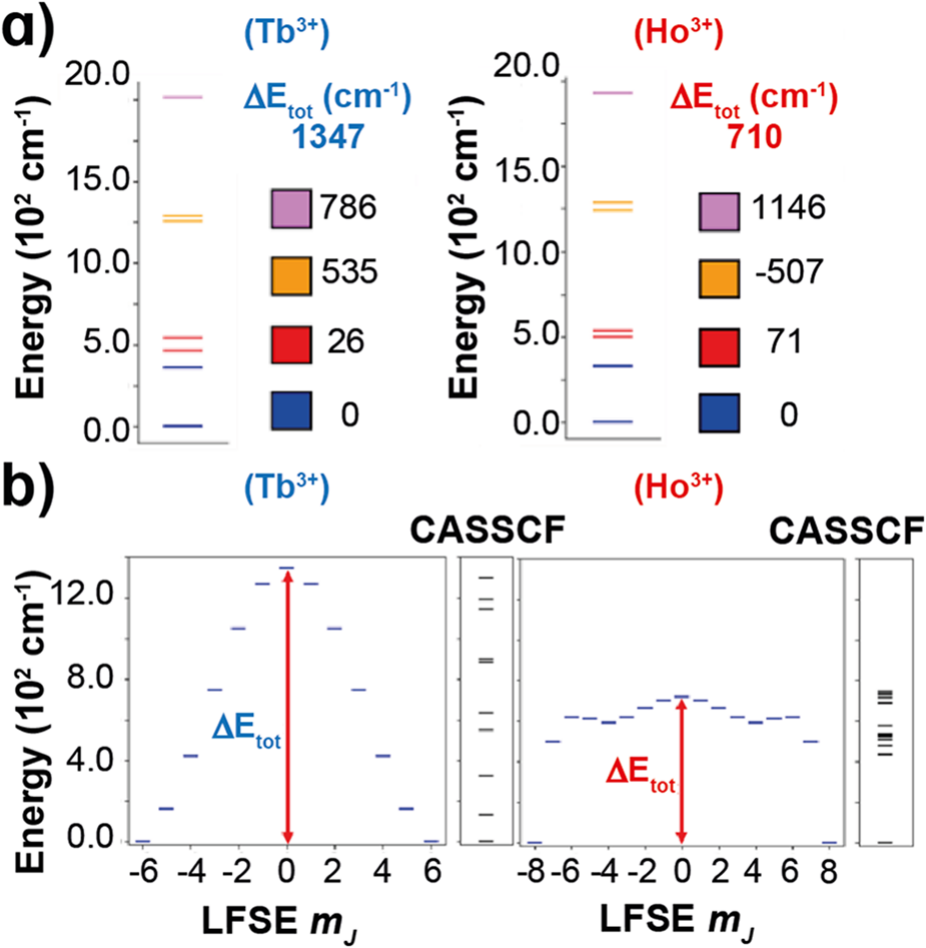
(a) 4f-orbital energy splitting obtained from ab initio ligand field (AILFT)^24^ calculations for the Tb^3+^ and Ho^3+^ complexes **3**·2CH_2_Cl_2_ and **4**·2CH_2_Cl_2_. Blue, red, orange, and violet correspond to the energy of the *f*_±3_, *f*_±2_, *f*_±1_, and f_0_ orbitals, respectively. The value for the barrier is shown in red (Δ*E*_tot_) and the contributions from each orbital block to the barrier are presented below. (b) LFSE (blue) and CASSCF (black) energies (cm^–1^) for the Tb^3+^ and Ho^3+^ complexes.

In the case of Ho^3+^, the ground state is separated from a dense block of excited states between 400 cm^–1^ and 750 cm^–1^ (see Figure 6b, right). This behavior can be understood considering that the Ho^3+^ ion presents an alternating pattern on the promotion/hampering of the anisotropy barrier. In this case, the most destabilized orbital (f_*z*_^3^) contributes 1146 cm^–1^ to the splitting (see Figure 6a, right) but the adjacent block (f_±1_) is detrimental (507 cm^–1^).

In the case of Tb^3+^, the total splitting is much higher (1347 cm^–1^) because all orbital blocks contribute positively due to the oblate-0 pattern of Tb^3+^. Despite this large splitting, the first excitation energy of the Tb^3+^ complex is rather small (130 cm^–1^ for CASSCF, Table 2, or 160 cm^–1^ for LFSE) because the energy difference between the *m*_J_ = ± 6 and *m*_J_ = ± 5 states depends on the difference between the average energy of the f_±3_ and f_±2_ blocks, which is only 319 cm^–1^ in this case.

**Table 2:** Energies (cm^–1^) for the States of the Ground Multiplet of the Tb^3+^ and Ho^3+^ Complexes, as Calculated by the CASSCF Method

Tb^3+^	Ho^3+^
0.00	0.00
0.04	0.03
130.06	430.10
130.18	435.47
321.14	473.79
323.36	475.42
547.47	506.53
629.81	521.27
883.57	528.96
895.68	531.84
1143.92	575.17
1192.15	684.50
1300.40	690.46
	714.90
	728.58
	733.24
	743.91

To complete this analysis, we employed the f-orbital energies calculated for Tb^3+^ to obtain the magnetic anisotropy of the previously published Dy^3+^ analogue (Figure S11).^7^ In this case, a large magnetic anisotropy is described, which is in line with the high-performance SMM properties exhibited for this compound.

These findings are consistent with the experimental observations. Despite the large total crystal field splitting observed for Tb^3+^ (1347 cm^–1^), the first excited state lies at just ∼130 cm^–1^, which significantly could limit its single-ion magnet behavior. This low-energy excitation arises from the relatively small energy gap between the f_±3_ and f_±2_ orbital blocks. In contrast, the Ho^3+^ complex displays a much denser distribution of excited states between 400 and 750 cm^–1^, providing a higher first excitation energy in comparison to that of Tb^3+^, despite its smaller overall splitting of the ground multiplet.

In the absence of an external magnetic field, neither complex exhibits SIM behavior, likely due to QTM arising from the splitting of the ground pseudodoublet (0.04 cm^–1^ for Tb^3+^ and 0.03 cm^–1^ for Ho^3+^, Table 2), which indicates that the ground state has some degree of mixing between m_J_ sublevels. The similarity in these energy splittings is consistent with the comparable experimental QTM relaxation times observed for **3**·2CH_2_Cl_2_ and **4**·2CH_2_Cl_2_ and explains the observed magnetic behavior.

## Conclusions

The hexagonal bipyramidal compounds {[Ln­(L^N6en^)­(OSiPh_3_)_2_]­(BPh_4_)}·2CH_2_Cl_2_ (Ln = Tb, **3**·2CH_2_Cl_2_; Ln = Ho, **4**·2CH_2_Cl_2_), which feature non-Kramers ions, do not exhibit SMM behavior under a zero applied magnetic field. This is attributed to the presence of QTM, which arises from the splitting of the ground pseudodoublet. The application of an optimal static field of 2000 Oe partially suppresses QTM, enabling both **3**·2CH_2_Cl_2_ and **4**·2CH_2_Cl_2_ to exhibit field-induced single-ion magnet (SIM) behavior, where magnetic relaxation occurs predominantly via QTM and Raman processes. At higher temperatures, the Raman mechanism becomes dominant, so the full temperature dependence of the relaxation time can be described by these two mechanisms. Although the overall magnetic performance is modest, these complexes represent the first examples of field-induced SIMs based on Tb^3+^ and Ho^3+^ ions with a hexagonal bipyramidal coordination environment. Moreover, they constitute the first reported Tb^3+^ and Ho^3+^ showing SIM behavior incorporating a macrocyclic ligand in a nonsandwich topology. Ab initio calculations support the experimental observations, revealing that the Ho^3+^ complex presents a significantly higher first excitation energy in comparison to Tb^3+^, which is interesting because Tb^3+^ shows a much higher splitting of its ground multiplet.

## Supplementary Material



## Notes

X-ray tables, crystal data and structure refinement, main bond distances and angles, IR spectra, ellipsoids diagram, PXRD, Cole–Cole plot, dependence of the relaxation time with the field, 4f-orbital energy splitting obtained from ab initio ligand field, and SHAPE calculations (PDF)
