# Supplementary material for: Geometry-Driven Field-Induced Single-Ion Magnetism in Hexagonal Bipyramidal Tb3+ and Ho3+ Complexes
Source: Inorg Chem. 2025 Oct 11;64(42):21088–97. doi: 10.1021/acs.inorgchem.5c03348 (PMC12570130; doi:10.1021/acs.inorgchem.5c03348)
Supplement: Supplementary file 1 [file ic5c03348_si_001.pdf]

Geometry-driven field-induced single-ion magnetism in hexagonal bipyramidal Tb^{3+} and Ho^{3+} complexes

Cristina González-Barreira,[‡] *Paula Oreiro-Martínez,*[‡] *Matilde Fondo,*[‡,]* *Julio Corredoira-Vázquez*[‡,ε,]* *Ana M. García-Deibe,*[‡] *Jesús Sanmartín-Matalobos,*[‡,ε] *Daniel Aravena*[§] *and Enrique Colacio*[£]

[‡] Departamento de Química Inorgánica, Facultade de Química, Universidade de Santiago de Compostela, 15782 Santiago de Compostela, Spain. E-mail: matilde.fondo@usc.es; julio.corredoira.vazquez@usc.es

[ε] Institute of Materials (iMATUS), Universidade de Santiago de Compostela, 15782 Santiago de Compostela, Spain

[§] Departamento de Química de los Materiales, Facultad de Química y Biología, Universidad de Santiago de Chile, Casilla 40, Correo 33 Santiago, Chile

[£] Departamento de Química Inorgánica, Facultad de Ciencias, Universidad de Granada, Avda Fuentenueva s/n, 18071 Granada, Spain

Corresponding Authors

Matilde Fondo, E-mail: matilde.fondo@usc.es

Julio Corredoira-Vázquez, E-mail: julio.corredoira.vazquez@usc.es

Table S1. Crystal data and structure refinement for **3**·$2CH_2Cl_2$ and **4**·$2CH_2Cl_2$. page S4

Table S2. Main bond distances (Å) and angles (°) for **3**·$2CH_2Cl_2$ and **4**·$2CH_2Cl_2$. page S5

Table S3. SHAPE v2.1. Continuous Shape Measures calculation (c) 2013. Electronic Structure Group, Universitat de Barcelona. page S6

Table S4. Tb^{3+} and Ho^{3+} complexes derived from N_6 macrocyclic ligands crystallographically characterized. page S7

Figure S1. Comparative IR spectra for: up) {[Tb(L^{N6en})($CH_3COO)_2$](BPh_4)}, **1** (blue) and {[Tb(L^{N6en})($OSiPh_3)_2$](BPh_4)}·$2CH_2Cl_2$, **3**·$2CH_2Cl_2$ (red). Bottom) {[Ho(L^{N6en})($CH_3COO)_2$](BPh_4)}·H_2O, **2**·H_2O (blue) and {[Ho(L^{N6en})($OSiPh_3)_2$](BPh_4)}·$2CH_2Cl_2$, **4**·$2CH_2Cl_2$ (red). page S8

Figure S2. Comparative IR spectra for: up) {[$Tb_{0.1}Y_{0.9}$(L^{N6en})($OSiPh_3)_2$](BPh_4)}·$1.5CH_2Cl_2$, **3@Y**·$1.5CH_2Cl_2$ (green) and {[Tb(L^{N6en})($OSiPh_3)_2$](BPh_4)}·$2CH_2Cl_2$, **3**·$2CH_2Cl_2$ (red). Bottom) {[$Ho_{0.1}Y_{0.9}$(L^{N6en})($OSiPh_3)_2$](BPh_4)}·CH_2Cl_2, **4@Y**·CH_2Cl_2 (green) and {[Ho(L^{N6en})($OSiPh_3)_2$](BPh_4)}·$2CH_2Cl_2$, **4**·$2CH_2Cl_2$ (red) page S9

Figure S3. a) Ellipsoids diagram for the cation [Tb(L^{N6en})($OSiPh_3)_2]^+$ in the {[Tb(L^{N6en})($OSiPh_3)_2$](BPh_4)} complex **3b1**, showing Tb2 as a polyhedron. Only heteroatoms of the asymmetric unit and those corresponding to the coordination sphere are labelled. The structure of **3b2** is very similar to that of **3b1**. b) N_6 equatorial environment of Tb2 in **3b1**, showing the deviation from the mean N_6 calculated plane. page S10

Figure S4. a) Ellipsoids diagram for the cation [Ho(L^{N6en})($OSiPh_3)_2]^+$ in the {[Ho(L^{N6en})($OSiPh_3)_2$](BPh_4)} complex **4b1**, showing the Ho^{3+} atom as a polyhedron. Only heteroatoms of the asymmetric unit and those corresponding to the coordination sphere are labelled. The structure of **4b2** is very similar to that of **4b1**. b) N_6 equatorial environment of Ho2 in **4b1**, showing the deviation from the mean N_6 calculated plane. page S11

Figure S5. Comparative powder X-ray diffractograms for: up) **3**·$2CH_2Cl_2$ (blue) and the simulation from single X-ray diffraction data (red). Bottom) **4**·$2CH_2Cl_2$ (blue) and the simulation from single X-ray diffraction data (red). page S12

Figure S6. $\chi_M T$ *vs* T and $M/N\mu_B$ *vs* H at 2 K for **3**·$2CH_2Cl_2$ (left) and **4**·$2CH_2Cl_2$ (right). The red lines represent the theoretical data obtained from ab initio calculations. page S13

Figure S7. Dependence of the relaxation time with the field for **3**·$2CH_2Cl_2$ at 5 K. The solid line is a guide for the eyes. page S13

Figure S8. Temperature dependence of χ''_M for **3**·$2CH_2Cl_2$ (a) and **4**·$2CH_2Cl_2$ (b) in a *dc* applied field of 2000 Oe at different frequencies. The solid lines are guides for the eyes. page S13

Figure S9. Cole–Cole plot for **3**·$2CH_2Cl_2$ (left) and **4**·$2CH_2Cl_2$ (right) in a *dc* applied field of 2000 Oe. The solid lines correspond to the best fits. page S14

Figure S10. Dependence of χ''_M with temperature under different fields at 10000 Hz for **3**·$2CH_2Cl_2$ (left) and **4**·$2CH_2Cl_2$ (right). The solid lines are guides for the eyes. page S14

Figure S11. Left) 4f-orbital energy splitting obtained from ab initio ligand field (AILFT) calculations for $[Dy(L^{N6en})(OSiPh_3)_2]^+$. Blue, red, orange and violet correspond to the energy of the $f_{\pm3}$, $f_{\pm2}$, $f_{\pm1}$, and f_0 orbitals, respectively. The value for the barrier is shown in red (ΔE_{tot}) and the contributions from each orbital block to the barrier are presented below. Right) LFSE (blue) energies (cm^{-1}). and CASSCF energies described in reference 1. page S15

References page S16

Table S1. Crystal data and structure refinement for **3**·$2CH_2Cl_2$ and **4**·$2CH_2Cl_2$.

	**3**·$2CH_2Cl_2$	**4**·$2CH_2Cl_2$
Empirical formula	$C_{80}H_{72}BCl_4N_6O_2Si_2Tb$	$C_{80}H_{72}BCl_4N_6O_2Si_2Ho$
Molecular weight	1517.14	1523.15
Crystal system	Triclinic	Triclinic
Space group	P-1	P1
Wavelength (Å)	0.71073	0.71073
Crystal size (mm^3)	0.100 x 0.080 x 0.020	0.100 x 0.060 x 0.030
Colour, shape	Colourless, plate	Colourless, plate
T (K)	100(2)	100(2)
a (Å)	14.636(2)	14.687(3)
b (Å)	15.556(2)	15.541(3)
c (Å)	16.400(3)	16.376(4)
α (º)	93.243(6)	93.205(7)
β (º)	97.561(6)	97.491(7)
γ (º)	105.130(5)	105.115(7)
Volume (Å^3)	3557.2(9)	3561.9(13)
Z	2	2
Absorpt. coef. (mm^{-1})	1.231	1.347
Reflections collected	124027	108413
Independent reflections	13029 [R_{int} = 0.1074]	29029 [R_{int} = 0.0542]
Data / restraints / param.	13029 / 6 / 988	29029 / 3214 / 1769
Final R indices [$I>2\sigma(I)$]	R_1 = 0.0424; wR_2 = 0.0910	R_1 = 0.0440; wR_2 = 0.0900
R indices (all data)	R_1= 0.0668; wR_2 = 0.1034	R_1= 0.0649; wR_2 = 0.1001

Table S2. Main bond distances (Å) and angles (°) for **3**·$2CH_2Cl_2$ and **4**·$2CH_2Cl_2$.

**3**·$2CH_2Cl_2$		**4**·$2CH_2Cl_2$	
Tb1-O1	2.156(3)	Ho1-O1	2.120(11)
		Ho1-O2	2.145(10)
Tb1-N11	2.643(4)	Ho1-N11	2.625(14)
		Ho1-N14	2.636(15)
Tb1-N12	2.665(4)	Ho1-N12	2.670(10)
		Ho1-N15	2.651(8)
Tb1-N13	2.674(3)	Ho1-N13	2.697(13)
		Ho1-N16	2.653(12)
Tb2-O2	2.155(3)	Ho2-O3; Ho2’-O3	2.193(11); 2.053(11)
		Ho2-O4; Ho2’-O4	2.096(11); 2.246(11)
Tb2-N21; Tb2-N21'	2.629(11); 2.705(13)	Ho2-N21; Ho2’-N21’	2.59(2); 2.68(3)
		Ho2-N24; Ho2’-N24’	2.65(3); 2.71(3)
Tb2-N22	2.666(4)	Ho2-N22; Ho2’-N22’	2.676(11); 2.65(2)
		Ho2-N25; Ho2’-N25’	2.637(13); 2.70(2)
Tb2-N23; Tb2-N23'	2.483(6); 2.775(8)	Ho2-N23; Ho2’-N23’	2.60(2); 2.61(3)
		Ho2-N26; Ho2’-N26’	2.60(2); 2.58(3)
O1-Tb1-O1#1	180.0	O1-Ho1-O2	178.6(6)
O1-Tb1-N11	97.10(11)	O1-Ho1-N11	95.5(4)
		O2-Ho1-N11	83.3(4)
O1-Tb1-N11#1	82.90(11)	O1-Ho1-N14	84.3(4)
		O2-Ho1-N14	96.8(4)
N11-Tb1-N11#1	180.0	N11-Ho1-N14	178.7(6)
N12-Tb1-N12#1	180.0	N12-Ho1-N15	178.9(4)
N13-Tb1-N13#1	180.0	N16-Ho1-N13	178.9(6)
N11-Tb1-N12	60.22(11)	N11-Ho1-N12	59.9(4)
N13-Tb1-N11#1	60.86(12)	N13-Ho1-N14	59.8(4)
N12-Tb1-N13	60.12(12)	N12-Ho1-N13	60.4(4)
O2-Tb2-O2#2	180.00(15)	O4-Ho2-O3	171.4(6)
O2-Tb2-N23	81.4(2)	O3-Ho2-N23	95.4(6)
		O4-Ho2-N23	78.7(6)
O2-Tb2-N23#2	98.6(2)	O3-Ho2-N26	103.1(6)
N21-Tb2-N21#2	180.0(4)	N21-Ho2-N24	164.8(9)
N22-Tb2-N22#2	180.0	N22-Ho2-N25	178.4(5)
N23-Tb2-N23#2	180.0	N23-Ho2-N26	161.3(8)
N23-Tb2-N21#2	53.8(3)	N23-Ho2-N24	63.6(7)
N22-Tb2-N23	62.9(2)	N23-Ho2-N22	57.6(6)
N21-Tb2-N22	63.5(3)	N21-Ho2-N22	63.2(6)
O2-Tb2-N21'#2	94.4(3)	O4-Ho2'-N23'	104.1(7)
O2-Tb2-N23'#2	79.11(19)	O3-Ho2'-N26'	79.4(7)
N21'-Tb2-N21'#2	180.0	N22'-Ho2'-N25'	176.8(6)
N22-Tb2-N21'	56.4(3)	N22'-Ho2'-N21'	58.2(7)
N22-Tb2-N23'	58.08(18)	N26'-Ho2'-N21'	59.6(8)
		O3-Ho2'-O4	168.5(6)

#1 -x,-y,-z+1; #2 -x+1,-y+1,-z

Table S3. SHAPE v2.1. Continuous Shape Measures calculation (c) 2013. Electronic Structure Group, Universitat de Barcelona.

ETBPY-8	13 D_{3h} Elongated trigonal bipyramid
TT-8	12 T_d Triakis tetrahedron
JSD-8	11 D_{2d} Snub diphenoid J84
BTPR-8	10 C_{2v} Biaugmented trigonal prism
JBTPR-8	9 C_{2v} Biaugmented trigonal prism J50
JETBPY-8	8 D_{3h} Johnson elongated triangular bipyramid J14
JGBF-8	7 D_{2d} Johnson gyrobifastigium J26
TDD-8	6 D_{2d} Triangular dodecahedron
SAPR-8	5 D_{4d} Square antiprism
CU-8	4 O_h Cube
HBPY-8	3 D_{6h} Hexagonal bipyramid
HPY-8	2 C_{7v} Heptagonal pyramid
OP-8	1 D_{8h} Octagon

3a

Structure [ML8]	ETBPY-8	TT-8	JSD-8	BTPR-8	JBTPR-8	JETBPY-8
	22.301,	7.513,	17.249,	16.421,	16.549,	24.191,
JGBF-8	TDD-8	SAPR-8	CU-8	**HBPY-8**	HPY-8	OP-8
10.714,	13.983,	16.753,	6.630,	**1.124**,	21.259,	29.214

3b1 (53.5%)

Structure [ML8]	ETBPY-8	TT-8	JSD-8	BTPR-8	JBTPR-8	JETBPY-8
	21.466,	9.308,	18.357,	17.202,	17.554,	24.485,
JGBF-8	TDD-8	SAPR-8	CU-8	**HBPY-8**	HPY-8	OP-8
10.620,	15.473,	18.269,	8.446,	**1.201**,	20.704,	27.917

3b2 (46.5%)

Structure [ML8]	ETBPY-8	TT-8	JSD-8	BTPR-8	JBTPR-8	JETBPY-8
	19.764,	8.578,	15.541,	15.567,	15.468,	21.100,
JGBF-8	TDD-8	SAPR-8	CU-8	**HBPY-8**	HPY-8	OP-8
9.383,	13.745,	16.372,	7.794,	**1.687**,	19.856,	27.991

4a

Structure [ML8]	ETBPY-8	TT-8	JSD-8	BTPR-8	JBTPR-8	JETBPY-8
	22.292,	7.531,	17.238,	16.174,	16.309,	24.238
JGBF-8	TDD-8	SAPR-8	CU-8	**HBPY-8**	HPY-8	OP-8
10.400,	13.769,	16.587,	6.663,	**1.171**,	21.110,	29.665

4b1 (52.5%)

Structure [ML8]	ETBPY-8	TT-8	JSD-8	BTPR-8	JBTPR-8	JETBPY-8
	19.722,	11.045,	15.005,	15.048,	15.323,	22.058,
JGBF-8	TDD-8	SAPR-8	CU-8	**HBPY-8**	HPY-8	OP-8
6.012,	14.383,	16.696,	10.247,	**2.009**,	21.014,	30.316

4b2 (47.5%)

Structure [ML8]	ETBPY-8	TT-8	JSD-8	BTPR-8	JBTPR-8	JETBPY-8
	20.772,	10.941,	15.393,	15.235,	15.616,	23.035,
JGBF-8	TDD-8	SAPR-8	CU-8	**HBPY-8**	HPY-8	OP-8
6.558,	14.260,	16.878,	10.286,	**1.850**,	20.249,	29.614

Table S4. Tb^{3+} and Ho^{3+} complexes derived from N_6 macrocyclic ligands crystallographically characterized.

Metal complex*	c.n./geom.[a]	Dy-N (Å)	Dy-O (Å)	Ref.
$[Tb(L)(NO_3)_2](NO_3)$	10/TD	2.574(3)- 2.633(2)	2.409(2)-2.465(2)	S1
$[Tb(L)(NO_3)_2](BPh_4)$	10/TD	2.592(4)- 2.641(3)	2.426(4)- 2.464(4)	S2
$[Tb(L)(NCS)_3]$	9/MFF	2.560(4)- 2.635(3)	-	S3
$[Tb_3L_3(\mu_2\text{-}F)_4(NO_3)_2](NO_3)_3$	10/TD-SSD	2.601(4)- 2.668(4)	2.528(3)- 2.530(3)	S4
$[Tb(L^1)(NCS)_3]$	9/MFF	2.568(3)-2.622(3)	-	S3
$[Tb(L^2)(NCS)_3]$	9/HH	2.598(6)-2.637(6)	-	S3
$[Tb(L^3)(CrO_4)(H_2O)](Cr_2O_7)_{0.5}$	9/MFF	2.529(8)- 2.655(9)	2.336(6)-2.367(8)	S5
$[Tb(L^3)(CH_3COO)_2](Cl)$	10/TD	2.569(2)- 2.657(2)	2.432(2)-2.485(2)	S6
$\{[Tb(L^{4R})Cl_2(MeOH)][Tb(L^{4R})Cl_2(H_2O)]\}(Cl)_2$	9/HH	2.562(4)- 2.632(3)	2.483(3)	S7
$[Tb(L^{5RII})(NO_3)(H_2O)](NO_3)_2$	9/MFF	2.534(3)- 2.613(6)	2.326(3)-2.458(6)	S8
$[Ho(L^{5RI})(NO_3)(H_2O)](NO_3)_2$	9/MFF	2.426(4)- 2.508(4)	2.418(4)-2.570(4)	S8

* Solvates are omitted; ligands in Scheme below. [a] TD: Tetradecahedron; SDD: Staggered Dodecahedron; MFF: muffin; HH: hula-hoop; CSAPR: Spherical capped square antiprism.

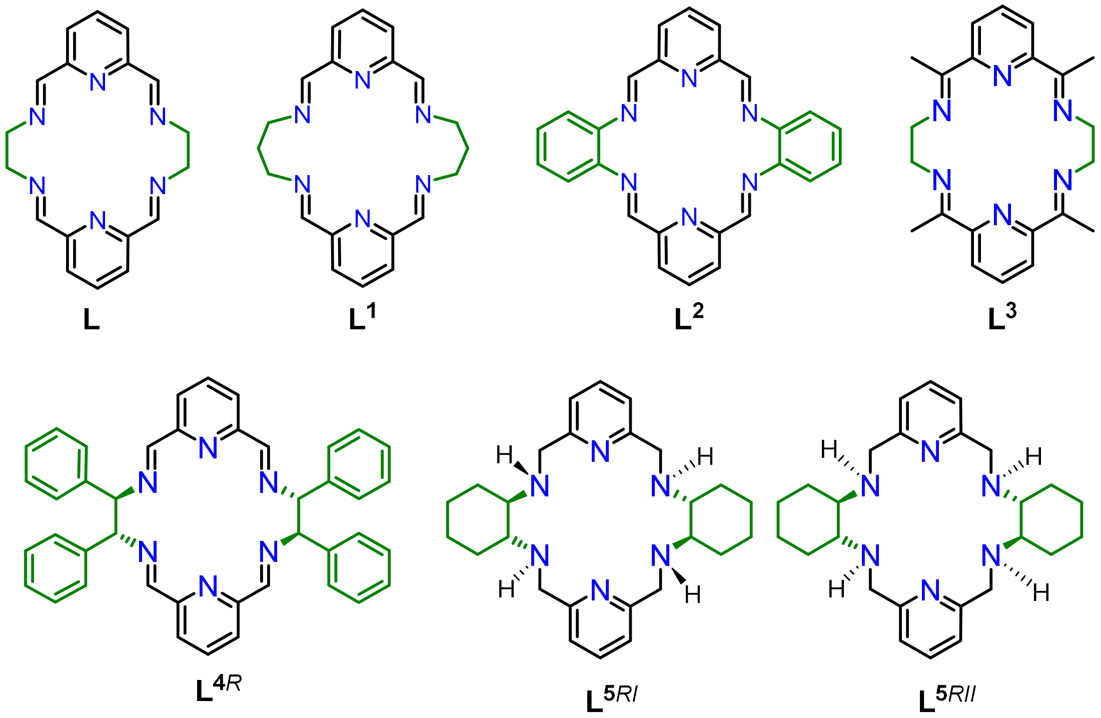

Ligands

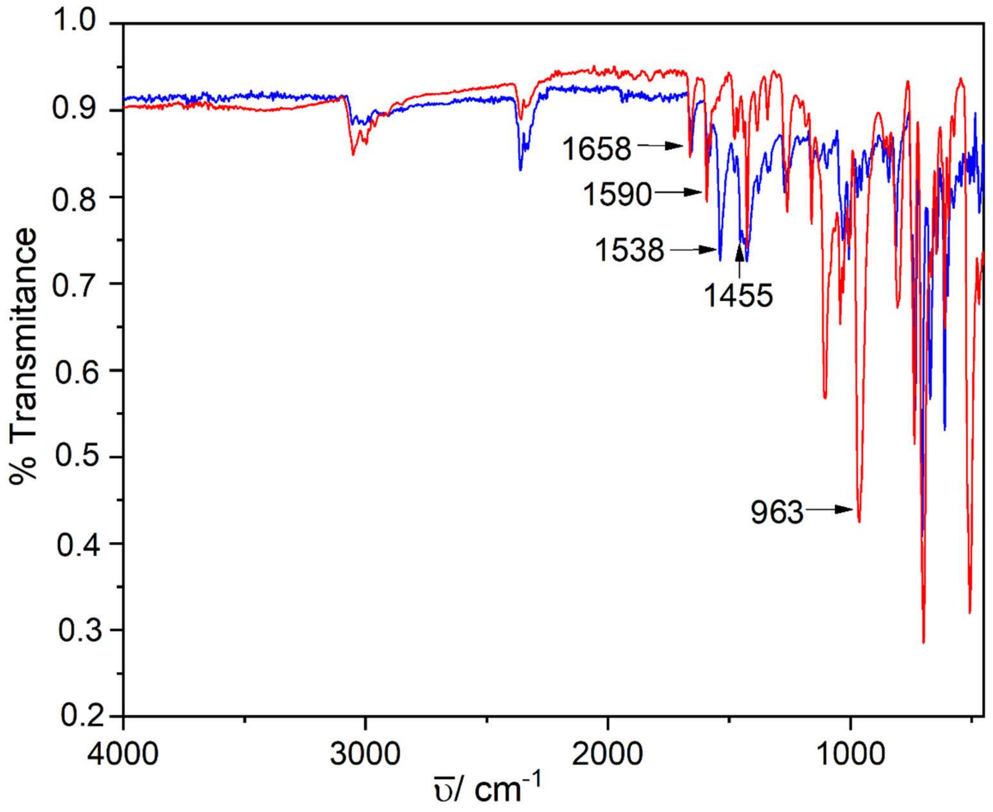

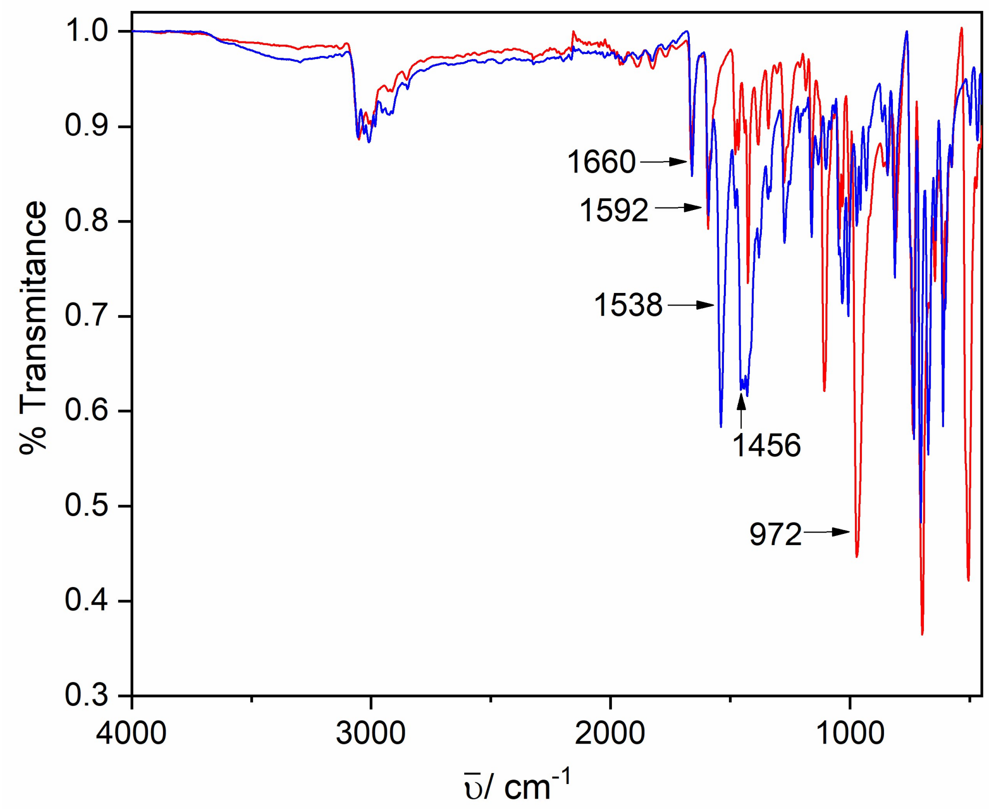

Figure S1. Comparative IR spectra for: up) {[Tb(L^{N6en})(CH_3COO)$_2$](BPh_4)}, **1** (blue) and {[Tb(L^{N6en})($OSiPh_3$)$_2$](BPh_4)}·2CH_2Cl_2, **3**·2CH_2Cl_2 (red). Bottom) {[Ho(L^{N6en})(CH_3COO)$_2$](BPh_4)}·H_2O, **2**·H_2O (blue) and {[Ho(L^{N6en})($OSiPh_3$)$_2$](BPh_4)}·2CH_2Cl_2, **4**·2CH_2Cl_2 (red).

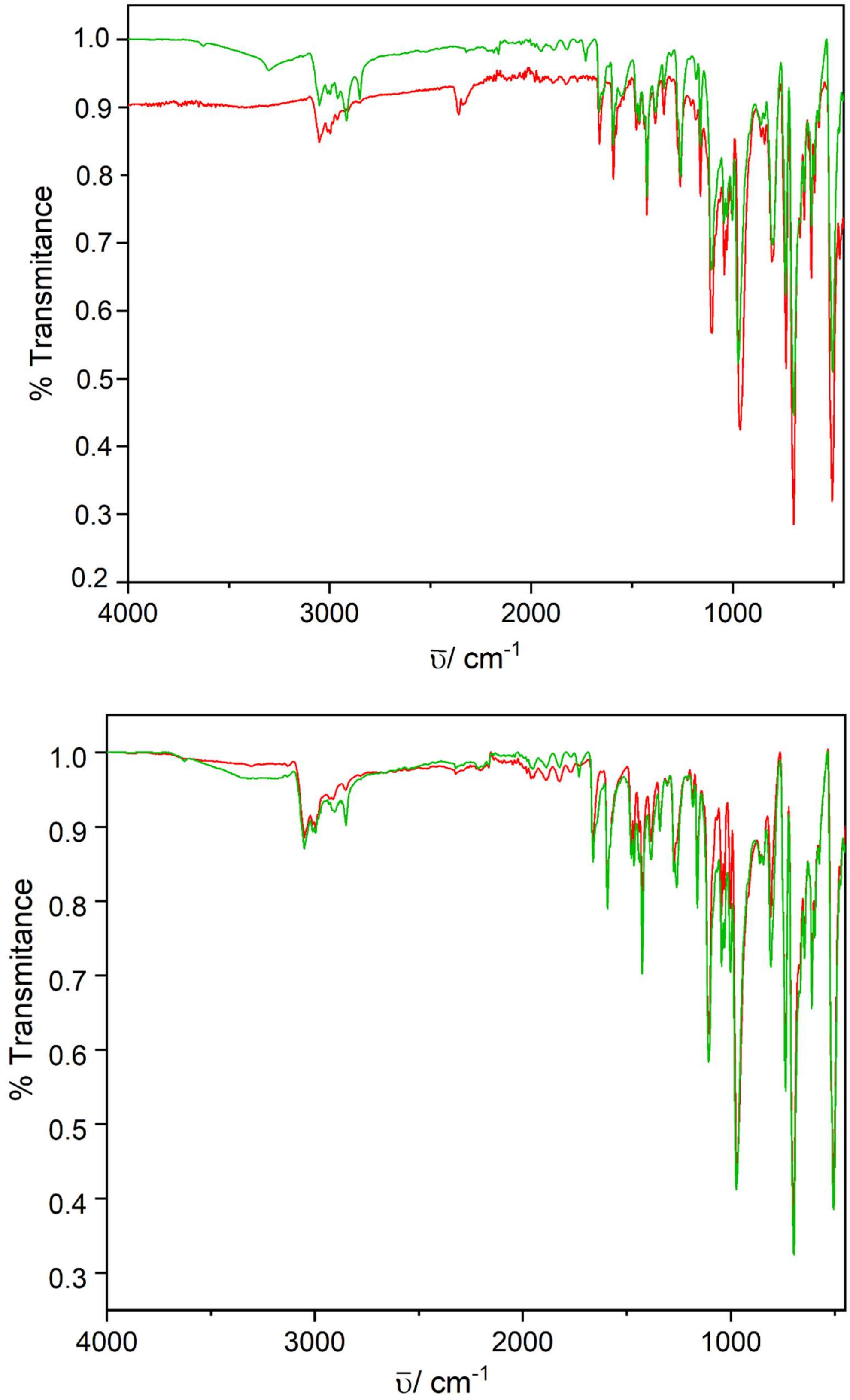

Figure S2. Comparative IR spectra for: up) $\{[Tb_{0.1}Y_{0.9}(L^{N6en})(OSiPh_3)_2](BPh_4)\}\cdot1.5CH_2Cl_2$, **3@Y**$\cdot1.5CH_2Cl_2$ (green) and $\{[Tb(L^{N6en})(OSiPh_3)_2](BPh_4)\}\cdot2CH_2Cl_2$, **3**$\cdot2CH_2Cl_2$ (red). Bottom) $\{[Ho_{0.1}Y_{0.9}(L^{N6en})(OSiPh_3)_2](BPh_4)\}\cdot CH_2Cl_2$, **4@Y**$\cdot CH_2Cl_2$ (green) and $\{[Ho(L^{N6en})(OSiPh_3)_2](BPh_4)\}\cdot2CH_2Cl_2$, **4**$\cdot2CH_2Cl_2$ (red).

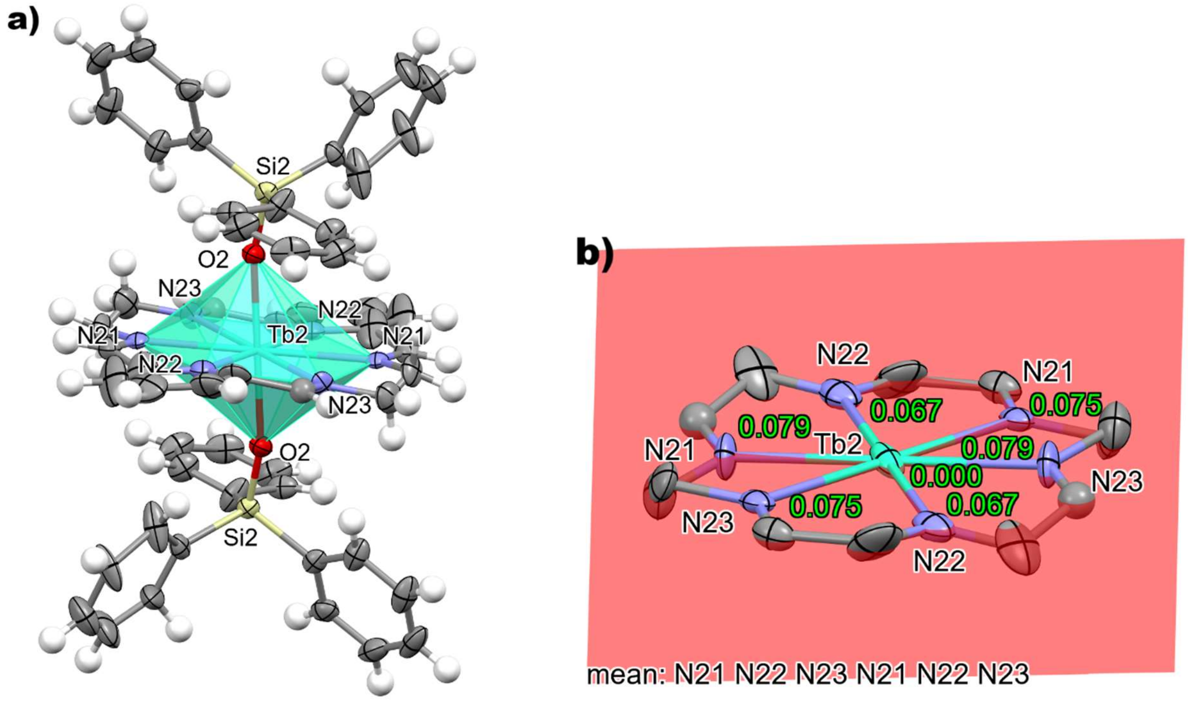

Figure S3. a) Ellipsoids diagram for the cation $[Tb(L^{N6en})(OSiPh_3)_2]^+$ in the $\{[Tb(L^{N6en})(OSiPh_3)_2](BPh_4)\}$ complex **3b1**, showing Tb2 as a polyhedron. Only heteroatoms of the asymmetric unit and those corresponding to the coordination sphere are labelled. The structure of **3b2** is very similar to that of **3b1**. b) N_6 equatorial environment of Tb2 in **3b1**, showing the deviation from the mean N_6 calculated plane.

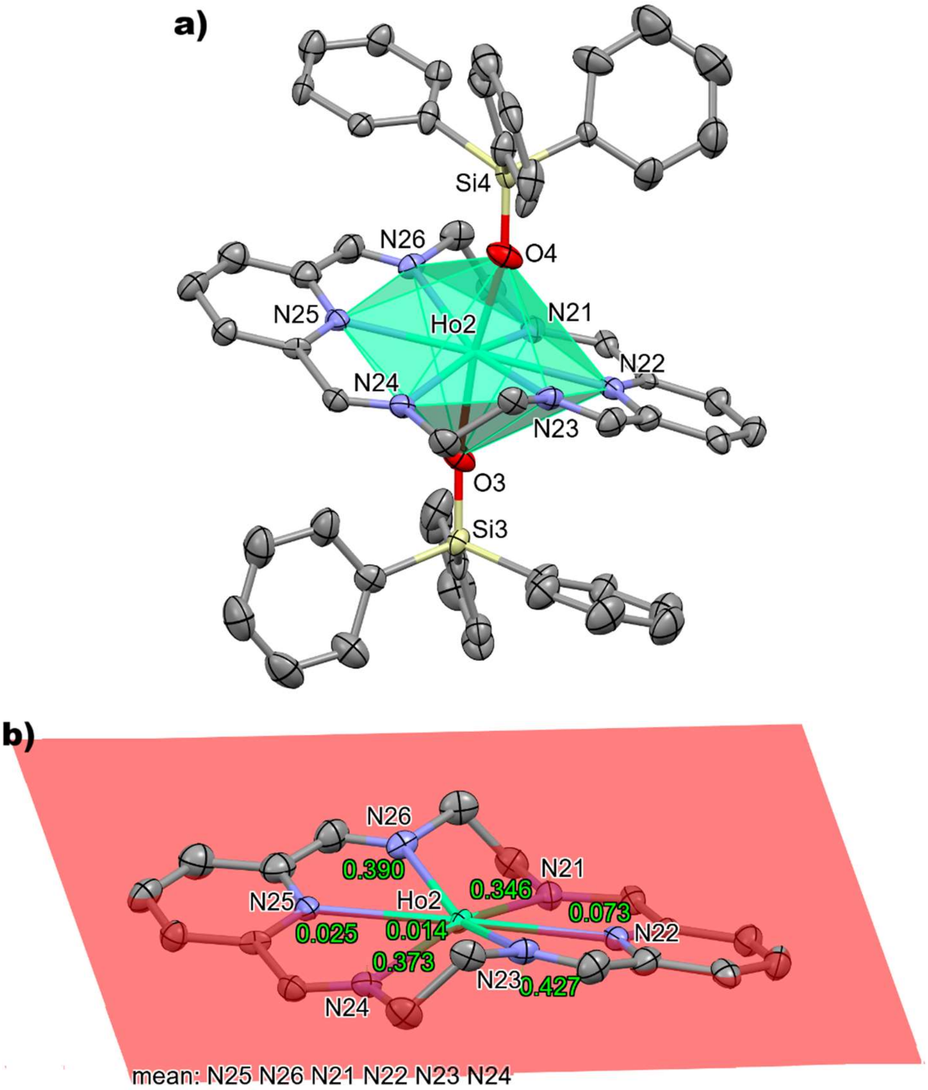

Figure S4. a) Ellipsoids diagram for the cation $[Ho(L^{N6en})(OSiPh_3)_2]^+$ in the $\{[Ho(L^{N6en})(OSiPh_3)_2](BPh_4)\}$ complex **4b1**, showing the Ho^{3+} atom as a polyhedron. Only heteroatoms of the asymmetric unit and those corresponding to the coordination sphere are labelled. The structure of **4b2** is very similar to that of **4b1**. b) N_6 equatorial environment of Ho2 in **4b1**, showing the deviation from the mean N_6 calculated plane.

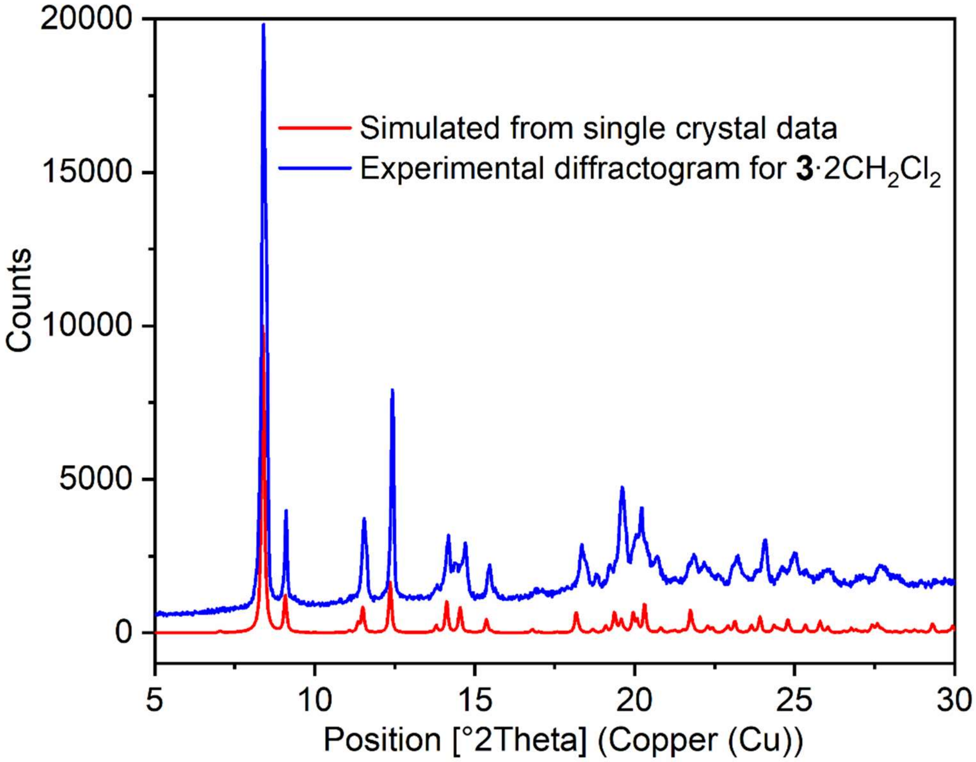

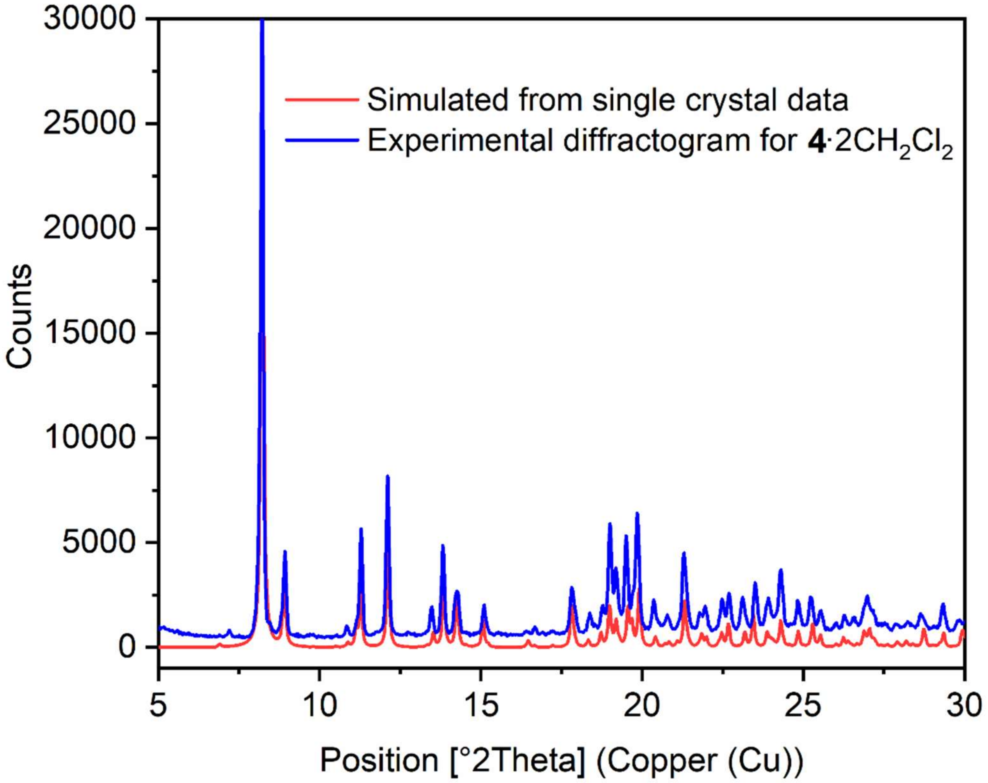

Figure S5. Comparative powder X-ray diffractograms for: up) **3**·$2CH_2Cl_2$ (blue) and the simulation from single X-ray diffraction data (red). Bottom) **4**·$2CH_2Cl_2$ (blue) and the simulation from single X-ray diffraction data (red).

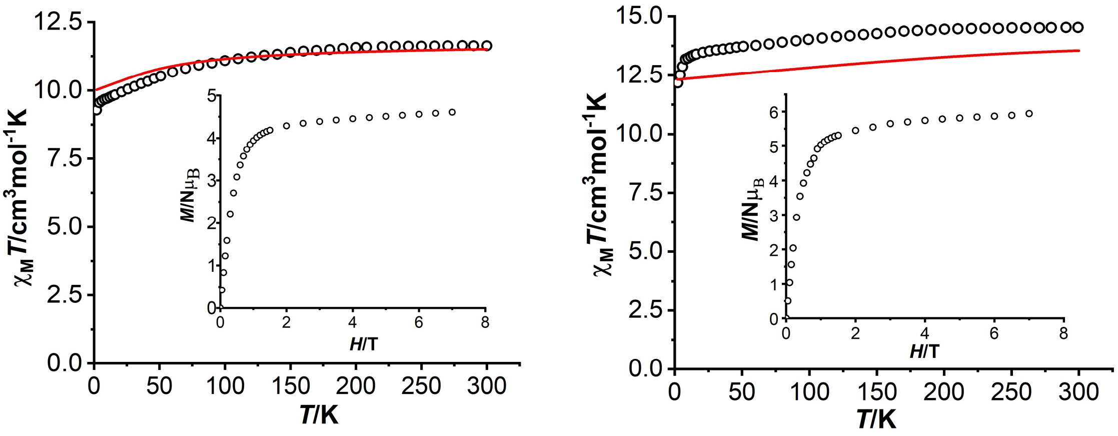

Figure S6. $\chi_M T$ *vs* T and $M/N\mu_B$ *vs* H at 2 K for **3**·$2CH_2Cl_2$ (left) and **4**·$2CH_2Cl_2$ (right). The red lines represent the theoretical data obtained from ab initio calculations.

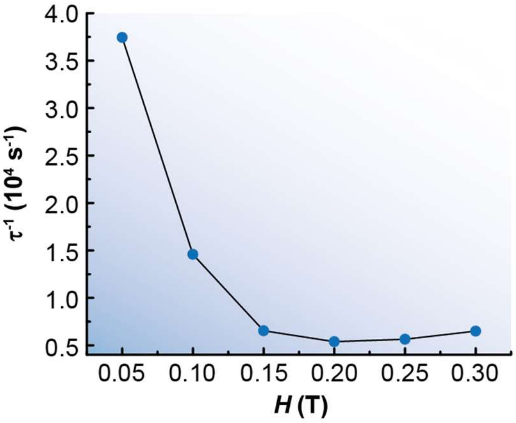

Figure S7. Dependence of the relaxation time with the field for **3**·$2CH_2Cl_2$ at 5 K. The solid line is a guide for the eyes.

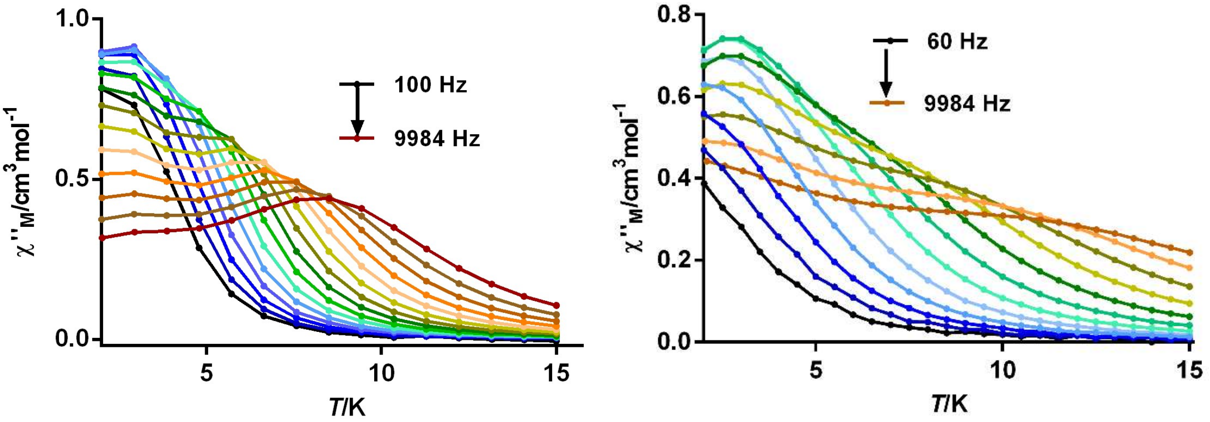

Figure S8. Temperature dependence of χ''_M for **3**·$2CH_2Cl_2$ (a) and **4**·$2CH_2Cl_2$ (b) in a *dc* applied field of 2000 Oe at different frequencies. The solid lines are guides for the eyes.

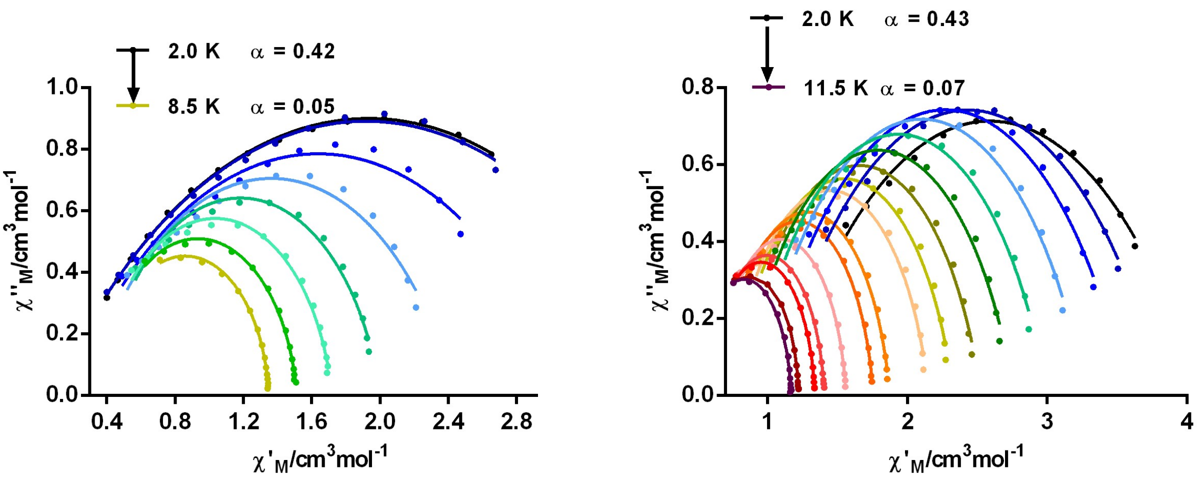

Figure S9. Cole–Cole plot for **3**·$2CH_2Cl_2$ (left) and **4**·$2CH_2Cl_2$ (right) in a *dc* applied field of 2000 Oe. The solid lines correspond to the best fits.

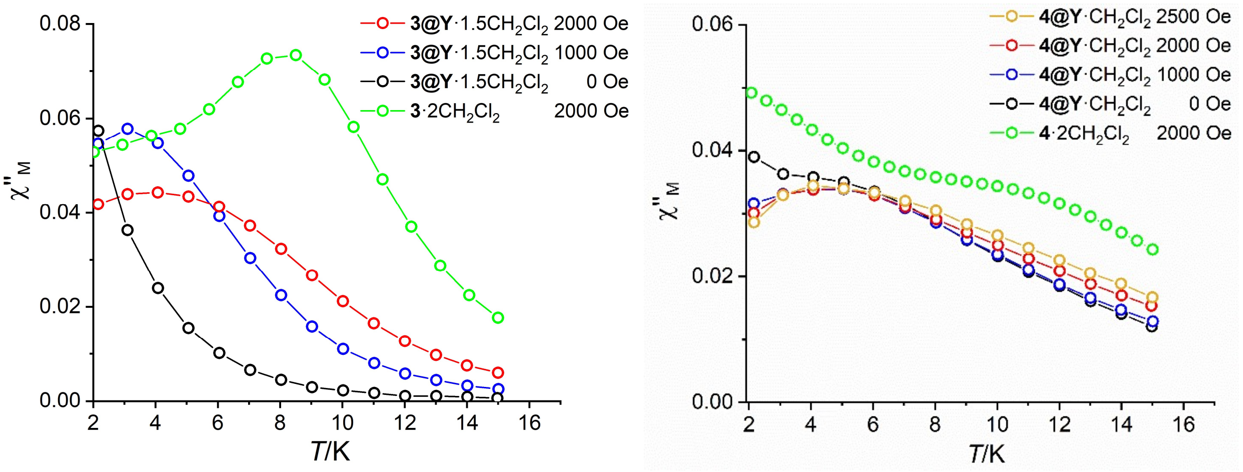

Figure S10. Dependence of χ''$_M$ with temperature under different fields at 10000 Hz for **3**·$2CH_2Cl_2$ (left) and **4**·$2CH_2Cl_2$ (right). The solid lines are guides for the eyes.

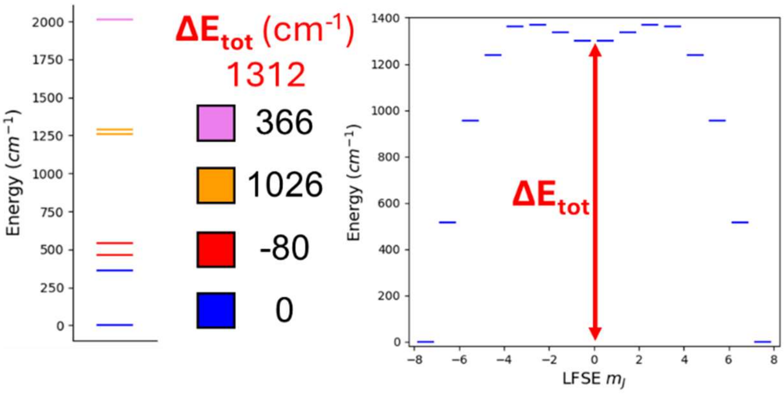

Figure S11. Left) 4f-orbital energy splitting obtained from ab initio ligand field (AILFT) calculations for $[Dy(L^{N6en})(OSiPh_3)_2]^+$. Blue, red, orange and violet correspond to the energy of the $f_{\pm3}$, $f_{\pm2}$, $f_{\pm1}$, and f_0 orbitals, respectively. The value for the barrier is shown in red (ΔE_{tot}) and the contributions from each orbital block to the barrier are presented below. Right) LFSE (blue) energies (cm^{-1}) described in reference S9.

REFERENCES

(S1) Villagra, D.; Fuentealba, P.; Spodine, E.; Vega, A.; Costa de Santana, R.; Verdejo, R.; Lopez-Manchado, M. A.; Aguilar-Bolados, H. Effect of terbium(III) species on the structure and physical properties of polyurethane (TPU). *Polymer* **2021**, *233*, 124209.

(S2) Liao, P.-Y.; Liu, Y.; Ruan, Z.-Y.; Wang, H.-L.; Shi, C.-G.; Deng, W.; Wu, S.-G.; Jia, J.-H.; Tong, M.-L. Magnetic and luminescent dual responses of photochromic hexaazamacrocyclic lanthanide complexes. *Inorg. Chem.* **2023**, *62* (3), 1075-1085.

(S3) Fuentealba, P.; Villagra, D.; Gil, Y.; Aguilar-Bolados, H.; Costa de Santana, R.; Gasparotto, G.; Vega, A.; Manzur, J.; Spodine, E. Thermal dependence of the luminescent properties of mononuclear Tb^{III} macrocyclic complexes. *Eur. J. Inorg. Chem.* **2021**, *2021* (44), 4543-4551.

(S4) Gawryszewska, P.; Ślepokura, K.; Lisowski, J. Triple-decker hexaazamacrocyclic lanthanide(III) complexes: structure, magnetic properties, and temperature-dependent luminescence. *Inorg. Chem.* **2024**, *63* (34), 15875-15887.

(S5) Ayala, J. D.; Bombieri, G.; Benetollo, F.; Gilli, P.; Vallarino, L. M. Structural and spectroscopic characterization of the macrocyclic complex: $[Tb(CrO_4)(H_2O)(C_{22}H_{26}N_6)]\cdot 0.5(Cr_2O_7)\cdot(H_2O)$. *J. Chem. Crystallogr.* **1995**, *25* (6), 355-360.

(S6) Voss, D. A.; Buttrey-Thomas, L. A.; Janik, T. S.; Rowen Churchill, M.; Morrow, J. R. Lanthanide(III) hexaaza macrocyclic Schiff-base complexes and their decomposition in aqueous solution. *Inorg. Chim. Acta* **2001**, *317* (1), 149-156.

(S7) Ślepokura, K.; Cabreros, T. A.; Muller, G.; Lisowski, J. Sorting Phenomena and chirality transfer in fluoride-bridged macrocyclic rare earth complexes. *Inorg. Chem.* **2021**, *60* (23), 18442-18454.

(S8) Gerus, A.; Ślepokura, K.; Lisowski, J. Anion and solvent induced chirality inversion in macrocyclic lanthanide complexes. *Inorg. Chem.* **2013**, *52* (21), 12450-12460.

(S9) Corredoira-Vázquez, J.; González-Barreira, C.; Fondo, M.; García-Deibe, A. M.; Sanmartín-Matalobos, J.; Gómez-Coca, S.; Ruiz, E.; Brites, C. D. S.; Carlos, L. D. An air-stable high-performance single-molecule magnet operating as a luminescent thermometer below its blocking temperature. *Inorg. Chem. Front.* **2025**, *12*, 5506-5512.
